# Application of Ion Mobility Spectrometry and the Derived Collision Cross Section in the Analysis of Environmental Organic Micropollutants

**DOI:** 10.1021/acs.est.3c03686

**Published:** 2023-12-13

**Authors:** Xue-Chao Song, Elena Canellas, Nicola Dreolin, Jeff Goshawk, Meilin Lv, Guangbo Qu, Cristina Nerin, Guibin Jiang

**Affiliations:** † School of the Environment, Hangzhou Institute for Advanced Study, University of the Chinese Academy of Sciences, Hangzhou 310024, China; ‡ State Key Laboratory of Environmental Chemistry and Ecotoxicology, Research Center for Eco-Environmental Sciences, Chinese Academy of Sciences, Beijing 100085, China; § Department of Analytical Chemistry, Aragon Institute of Engineering Research I3A, EINA, University of Zaragoza, Maria de Luna 3, 50018 Zaragoza, Spain; ∥ Waters Corporation, Stamford Avenue, Altrincham Road, SK9 4AX Wilmslow, United Kingdom; ⊥ Research Center for Analytical Sciences, Department of Chemistry, College of Sciences, Northeastern University, 110819 Shenyang, China; # Institute of Environment and Health, Jianghan University, Wuhan 430056, China

**Keywords:** ion mobility, collision cross section, environmental organic micropollutants, suspect screening, nontargeted analysis

## Abstract

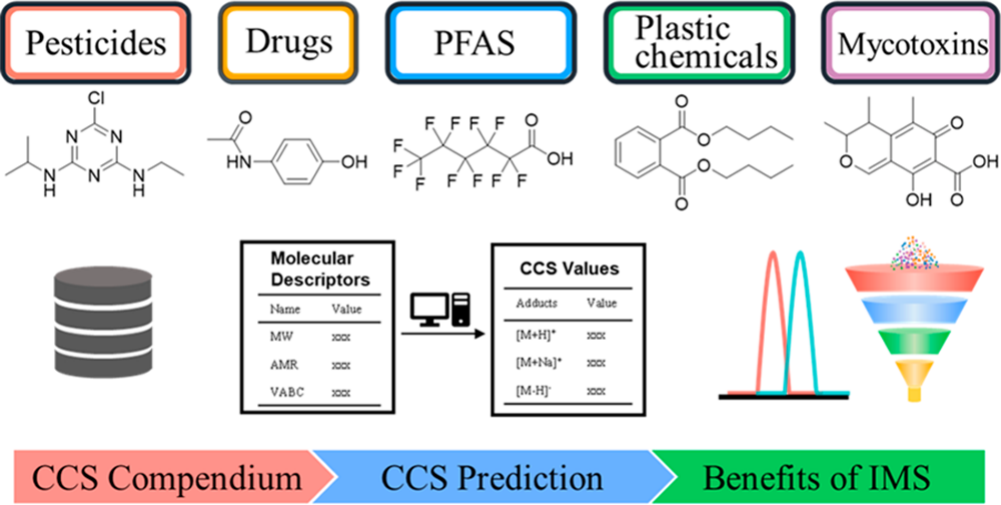

Ion mobility spectrometry (IMS) is a rapid gas-phase separation technique, which can distinguish ions on the basis of their size, shape, and charge. The IMS-derived collision cross section (CCS) can serve as additional identification evidence for the screening of environmental organic micropollutants (OMPs). In this work, we summarize the published experimental CCS values of environmental OMPs, introduce the current CCS prediction tools, summarize the use of IMS and CCS in the analysis of environmental OMPs, and finally discussed the benefits of IMS and CCS in environmental analysis. An up-to-date CCS compendium for environmental contaminants was produced by combining CCS databases and data sets of particular types of environmental OMPs, including pesticides, drugs, mycotoxins, steroids, plastic additives, per- and polyfluoroalkyl substances (PFAS), polycyclic aromatic hydrocarbons (PAHs), polychlorinated biphenyls (PCBs), and polybrominated diphenyl ethers (PBDEs), as well as their well-known transformation products. A total of 9407 experimental CCS values from 4170 OMPs were retrieved from 23 publications, which contain both drift tube CCS in nitrogen (^DT^CCS_N_2__) and traveling wave CCS in nitrogen (^TW^CCS_N_2__). A selection of publicly accessible and in-house CCS prediction tools were also investigated; the chemical space covered by the training set and the quality of CCS measurements seem to be vital factors affecting the CCS prediction accuracy. Then, the applications of IMS and the derived CCS in the screening of various OMPs were summarized, and the benefits of IMS and CCS, including increased peak capacity, the elimination of interfering ions, the separation of isomers, and the reduction of false positives and false negatives, were discussed in detail. With the improvement of the resolving power of IMS and enhancements of experimental CCS databases, the practicability of IMS in the analysis of environmental OMPs will continue to improve.

## 1 Introduction

Synthetic chemicals are extensively used in everyday life and these chemicals which include pesticides, pharmaceuticals, and personal care products (PPCPs), together with industrial chemicals, can enter the environment and pose a potential threat to human health and the ecological environment.^1,2^ A large number of publications have proven that some of these synthetic chemicals can disrupt the endocrine system and contribute to reproductive disorders, allergic diseases, and even cancer.^3−5^ Thus, it is important to monitor the occurrence and distribution of these chemicals in various environmental systems.

Gas chromatography (GC) and liquid chromatography (LC) coupled to high-resolution mass spectrometry (HRMS) are the instruments most used for analyzing environmental samples for the presence of chemicals.^6^ In recent years, with the development of data processing software and related databases, HRMS-based suspect screening analysis (SSA) and nontargeted analysis (NTA) have been widely used for the chemical characterization of complex environmental samples, such as indoor dust, sediment, and airborne particulate matter.^7−10^ However, the high complexity of sample matrices, the presence of isomers, and low concentrations of contaminants bring analytical challenges to the current SSA or NTA workflow. The addition of an extra separation dimension to the conventional GC– or LC–HRMS systems provides the possibility of being able to alleviate some of these analytical challenges.^11−13^

Ion mobility spectrometry (IMS) is a rapid gas-phase separation technique (normally on the millisecond time scale), which has received growing interest from researchers in the last decades. The basic principle of IMS is the separation of ions in buffer gas under the influence of an electric field.^13^ In temporally dispersive IMS devices, ions with smaller cross sections move faster than ions with larger cross sections in the drift cell, as the former have less interaction with buffer gas. Since IMS allows the separation of ions on the basis of their shape, size and charge, it can provide complementary structural information to add to those of retention time (RT) and *m*/*z*. Since the first commercial LC–IMS–MS platform, Synapt HDMS, was introduced by Waters Corporation in 2006,^14^ a flourishing growth of the use of IMS in the characterization of small molecules has been observed. This growth has encompassed various chemicals including metabolites,^15,16^ phenolic compounds,^17−19^ food and environmental contaminants,^20−23^ and extractables and leachables (E&L) from the pharmaceutical or food industries.^24−27^ The coupling of IMS with LC–HRMS has proven to be a powerful tool for the separation and identification of small molecules.

The IMS-derived collision cross section (CCS) is a stable physiochemical parameter of ionized molecules, which can serve as additional identification evidence in unknown characterization.^13^ Unlike RT which varies with experimental conditions, CCS measurements are independent from sample matrix and chromatographic and mass spectrometric conditions,^28,29^ with high reproducibility across different platforms and laboratories.^30−32^ These characteristics of CCS make it a reliable parameter for inclusion in the process of unknown identification. So far, several CCS databases have been built for environmental contaminants, including pesticides,^33^ pharmaceuticals,^34^ and industrial chemicals.^20,35^ However, a comprehensive CCS compendium for environmental organic micropollutants (OMPs) is still not available.

In 2017^36^ and 2019,^37^ the McLean laboratory presented two CCS compendiums to aid the multiomics compound identities, which were focused on the CCS values of biomolecules. In the study of May and co-workers,^36^ 1477 experimental CCS values of small molecules (hydrocarbons and metabolites) were included in the final compendium. Given that the CCS values of a large number of environmental contaminants were reported after 2019, in this review, we have produced an up-to-date CCS compendium for environmental contaminants by combining both drift tube CCS in nitrogen (^DT^CCS_N_2__) and traveling wave CCS in nitrogen (^TW^CCS_N_2__) databases and data sets of particular types of environmental OMPs, including pesticides, drugs, mycotoxins, steroids, plastic additives, per- and polyfluoroalkyl substances (PFAS), polycyclic aromatic hydrocarbons (PAHs), polychlorinated biphenyls (PCBs), and polybrominated diphenyl ethers (PBDEs), as well as their well-known transformation products. We also introduce a selection of publicly accessible and in-house CCS prediction tools and discuss the factors affecting CCS prediction accuracy. Lastly, we summarize the advances in utilizing IMS in the analysis of various types of OMPs and describe the benefits of IMS in targeted analysis, SSA, and NTA.

In 2011, Marquez-Sillero and co-workers summarized the use of IMS in the targeted analysis of environmental contaminants; however, they did not publish any CCS values.^38^ As IMS techniques have evolved rapidly over recent years and a large amount of CCS data with respect to environmental OMPs has been reported in the past decade, a review summarizing the advances of IMS in the field of environmental analysis is necessary. We hope this work can facilitate the application of IMS in the screening and identification of OMPs in complex environmental matrices.

## 2 Basic Principles of IMS and Different Instrumentation

The origin of IMS can be traced back to 1896, when the mobility of ions in various gases was investigated by Thomson and Rutherford.^39^ The fundamental principle of IMS is “packets of gas-phase ions pass through a drift tube filled with buffer gas under the influence of a weak electric field (*E*)”.^13^ As IMS provides complementary information to RT and mass spectra, the combination of IMS with LC–MS systems has attracted increasing interest from researchers in the analysis of complex mixtures. The most commonly used IMS techniques in environmental analyses are drift tube IMS (DTIMS), traveling wave IMS (TWIMS), and trapped IMS (TIMS). There are also other types of IMS techniques, such as field asymmetric waveform ion mobility spectrometry (FAIMS) and differential mobility analyzer (DMA); however, these techniques are not discussed in this review due to their relatively low usage in environmental analysis. A brief introduction of DTIMS, TWIMS, and TIMS is given in the Supporting Information, and more detailed introductions of each of the IMS devices can be obtained in specialized literature.^40−44^

## 3 CCS Compendium for Environmental Organic Micropollutants

CCS has been demonstrated to be extremely reproducible across different instrumentations and laboratories. The study of Righetti et al.^32^ indicated that the ^TW^CCS_N_2__ measurements between two Vion IMS platforms from different laboratories showed deviations of less than 1.5%. Additionally, 96.4% of ^TW^CCS_N_2__ values measured on Vion and Synapt platforms have deviations within 2%.^32^ An average of 0.29% relative standard deviation (RSD) and an average absolute bias of 0.54% were also observed for ^DT^CCS_N_2__ in an interlaboratory study.^45^ In addition, Hinnenkamp et al.^31^ compared the CCS values determined by TWIMS and DTIMS, finding that 93% of [M + H]^+^ ions and 87% of [M + Na]^+^ ions had CCS deviations lower than 2%. The high reproducibility of CCS makes it a reliable molecular identifier that can be incorporated into HRMS-based screening workflows. Currently, there are several experimental CCS databases and data sets available for different types of OMPs, such as pesticides, drugs, mycotoxins, steroids, and plastic additives. Table 1 introduces 23 open-access experimental CCS databases and data sets related to the OMPs.

**Table 1:** CCS Values of Emerging Contaminants Published between 2016 and 2022^a^

compound type	year	type	number of CCS	remarks	ref
pesticides	2016	^TW^CCS_N_2__	214	CCS was measured in positive electrospray ionization mode; some CCS values were from the in-source fragments of molecules	(33)
pesticides	2017	^TW^CCS_N_2__	205	only the CCS values of protonated molecules were measured	(46)
pesticides	2022	^TW^CCS_N_2__	110	only the CCS values of protonated molecules were measured	(47)
drug-like compounds and pesticides	2016	^DT^CCS_N_2__	61	more than 500 standards including drug-like compounds and pesticides were detected; only a limited number of CCS values were disclosed	(48)
drug or drug-like molecules	2017	^TW^CCS_N_2__	1416	CCS values of multiply charged ions were not retrieved	(34)
pharmaceuticals, drugs of abuse, and their metabolites	2018	^TW^CCS_N_2__	357	only the CCS values of protonated molecules were measured	(49)
human and veterinary drugs	2018	^TW^CCS_N_2__	173	CCS values were measured under positive ionization conditions	(50)
abused drugs and toxic compounds	2018	^DT^CCS_N_2__	124	only the CCS values of protonated molecules were measured	(51)
drugs	2021	^TW^CCS_N_2__	3225	CCS values of both cations and anions	(52)
antiepileptic drugs	2020	^DT^CCS_N_2__	13	only the CCS values of protonated molecules were measured	(53)
pharmaceutical metabolites	2021	^TW^CCS_N_2__	10	CCS values of 8 [M + H]^+^ ions and 2 [M – H]^−^ ions	(54)
doping agents	2021	^TW^CCS_N_2__	176	CCS values were measured under positive ionization conditions	(55)
opioids	2022	^DT^CCS_N_2__	33	only the CCS values of protonated molecules were provided	(56)
PAHs, PCBs, PBDEs, and their metabolites	2018	^DT^CCS_N_2__	138	compounds ionized by different ion sources	(57)
pesticides, PAHs, PCBs, flame retardants	2022	^TW^CCS_N_2__	236	some CCS values were not disclosed in Supporting Information	(58)
PFAS, PAHs, PCBs, PBDEs	2022	^DT^CCS_N_2__	202	PAHs were detected in positive ionization mode; other compounds in negative ion mode	(59)
PFAS	2020	^DT^CCS_N_2__	39	CCS values were measured in negative mode	(22)
mycotoxins	2020	^TW^CCS_N_2__	207	interlaboratory and interplatform reproducibility were evaluated	(32)
steroids	2020	^TW^CCS_N_2__	173	interlaboratory and interplatform reproducibility were evaluated	(30)
pollutants in indoor dust: flame retardants, pesticides	2020	^TW^CCS_N_2__	45	CCS values of some adducts were not disclosed	(60)
CECs in human matrix, including bisphenols, plasticizers, OPFRs, and triazoles	2021	^DT^CCS_N_2__	240	71 CCS values of dimers were not retrieved	(20)
chemicals in plastic food packaging, including antioxidants, plasticizers, UV absorbers, lubricants, and NIAS	2022	^TW^CCS_N_2__	1056	1038 CCS published values with 18 newly measured CCS values	(35)
organic environmental pollutants, including illicit drugs, hormones, mycotoxins, new psychoactive substances, pesticides, and pharmaceuticals	2020	^TW^CCS_N_2__	956	970 different adducts from 556 compounds	(21)

a Abbreviations: PFAS, per- and polyfluoroalkyl substances; PAHs, polycyclic aromatic hydrocarbons; PCBs, polychlorinated biphenyls; PBDEs, polybrominated diphenyl ethers; CECs, contaminants of emerging concern; NIAS, non-intentionally added substances; OPFRs, organophosphate flame retardants.

### 3.1 CCS Data Collection

A total of 9407 experimental CCS values from 4170 OMPs were retrieved from 23 scientific articles published in the period from 2016 to 2022, as shown in Table 1. All of the CCS measurements were conducted with nitrogen as the buffer gas. Figure S1 shows the distribution of CCS values published over time as well as the contributions from the main research groups. Approximately 70% (6719) of the CCS values related to environmental OMPs were reported between 2020 and 2022, which may be due to the increasing popularity of adopting commercially available IMS-HRMS platforms for routine chemical analysis. More details about these 23 publications can be found in Table 1.

There are some publications in which the measured CCS values were not fully disclosed. For example, the work of Stephan and co-workers investigated the CCS values for more than 500 standard substances including drug-like molecules and pesticides; however, only 61 CCS values were reported in the final publication.^48^ The study from Izquierdo and co-workers states that 202 CCS values for [M + H]^+^ and 168 for [M]^+^* were measured for PAHs, PCBs, flame retardants, and pesticides; however, only 145 CCS values for [M + H]^+^ and 91 CCS values for [M]^+*^ were finally reported.^58^ The undisclosed CCS data could limit the chemical space of this CCS compendium.

**Figure 1:**
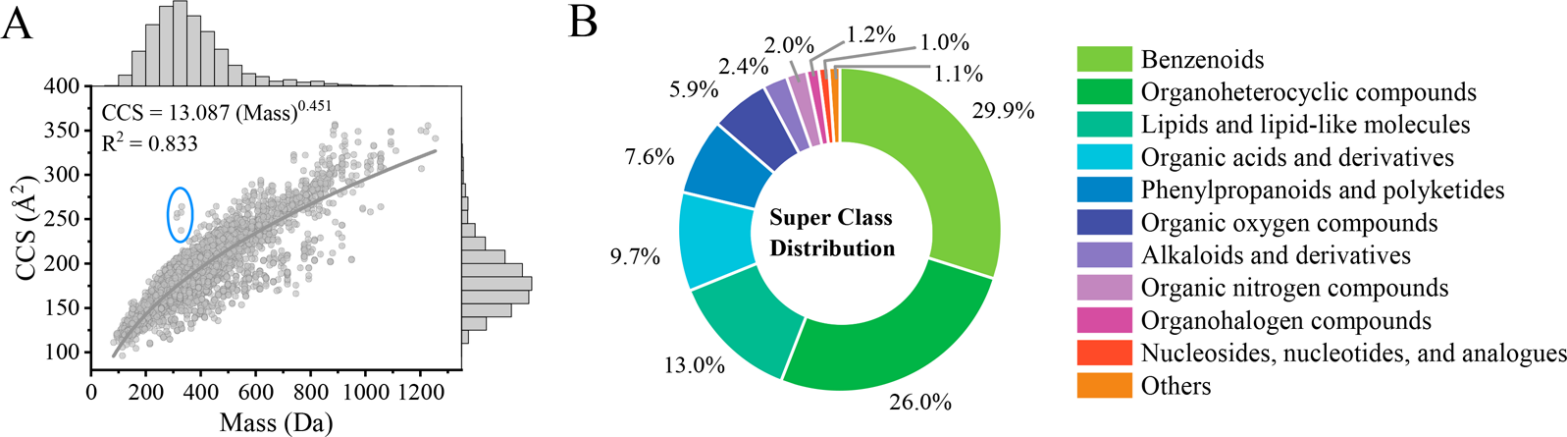
(A) Depiction of CCS values vs mass values for 9407 ions together with the distribution of CCS and *m*/*z* values. (B) Distribution of 4170 compounds across super classes.

The correlation between CCS values and mass for 9407 ions and the distribution of CCS and mass values are shown together in Figure 1A. The relationship between CCS and mass was described by the power regression model; with *R*^2^ of 0.833, the CCS compendium ranged in molecular weight from 82 (fomepizole) to 1255 Da (dactinomycin), with CCS values ranging from 96.5 Å^2^ ([M – H – CO_2_]^−^ of trifluoroacetic acid) to 357.3 Å^2^ ([M + H]^+^ of ledipasvir). Five ions clearly located out of the trend lines are highlighted in the blue circle; these ions belong to the [M + NH_4_]^+^ adducts of mycotoxins and were collected from the study of Righetti et al.^32^ These CCS values need to be examined considering their significantly high deviations (∼90 Å^2^) with the CCS values of corresponding [M + H]^+^ ions. The super classes of the 4170 compounds in the compendium were obtained using ClassyFire,^61^ and the distribution of compounds across super classes is shown in Figure 1B and Table S1. The compendium covers 17 super classes, although 7 of the super classes contain only a few compounds, with proportions lower than 0.5%; thus, these 7 super classes are not depicted separately in Figure 1B. Most of the compounds in the compendium belong to the benzenoids class (29.9%), which consists of a variety of contaminants, including pesticides, drugs, PAHs, phthalate plasticizers, and other plastic additives. Organoheterocyclic compounds (26.0%) contain many pesticides and drug-like compounds. Lipids and lipid-like molecules (13.0%) and organic acids and derivatives (9.7%) also represent a large part of the compendium; the former mainly contains steroids and non-phthalate plasticizers, and the latter includes drugs, OPFRs, and PFAS. Our compendium showed a different composition than that of the compendium curated by Picache and co-workers,^37^ with the latter mainly containing lipid and lipid-like molecules and organic acids and derivatives and only 7% of the compendium belonging to the super class of benzenoids. This highlights the need for a specialized CCS compendium for environmental OMPs. This specialized CCS compendium can benefit the screening analysis of OMPs in environmental samples and facilitate the development of an accurate CCS prediction model for OMPs.

### 3.2 CCS Distributions

#### 3.2.1 CCS from DTIMS versus TWIMS

**Figure 2:**
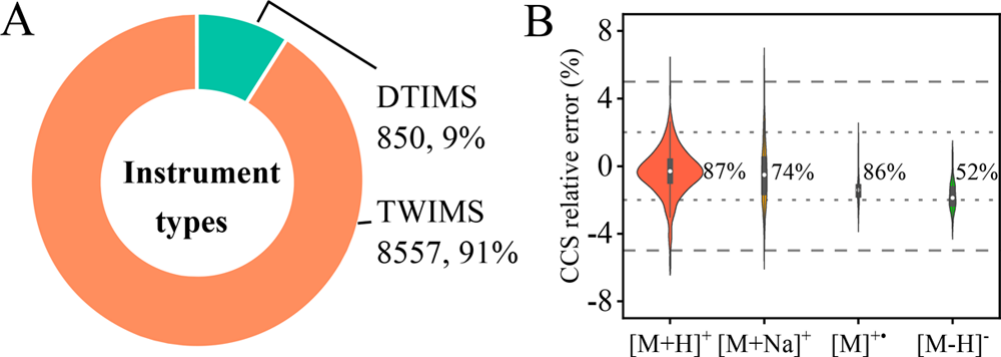
Different types of CCS values (A) and their relative errors (B).

The distribution of measured CCS values across different IMS instruments is shown in Figure 2A. Approximately 91% (8557) of the CCS values were measured using TWIMS devices, and the other 9% of CCS values (850) were measured using DTIMS instruments. Of these 850 ^DT^CCS_N_2__ values, 138 were measured by the stepped field method and 712 were measured by the single field method. The CCS compendium proposed by May and co-workers in 2017 has a totally different composition, with 87% of the CCS values measured using DTIMS instruments over the years from 1975 to 2015.^36^ The high number of CCS values measured by TWIMS since 2016 can be attributed to the introduction of the Vion IMS-quadrupole time-of-flight mass spectrometry (IMS-QTOF) by Waters Corporation in 2015. In this CCS compendium, 3353 CCS values from nine publications were determined using a Vion IMS-QTOF,^21,33,35,46,47,49,55,58,60^ and the CCS databases for steroids and mycotoxins were developed using both the Vion and Synapt platforms.^30,32^

A comparison of the empirical CCS values measured via DTIMS and TWIMS devices was performed, and the CCS deviations were calculated using the corresponding ^DT^CCS as reference values. A total of 580 CCS deviations were obtained, including 480 deviations from [M + H]^+^ ions, 38 from [M + Na]^+^ ions, 14 from [M]^+^* ions, and 48 from [M – H]^−^ ions. A more detailed distribution of the CCS deviations is shown in Table S2 and Figure 2B. It should be noted that in some cases multiple CCS deviations can be calculated for each ion; for example, two ^DT^CCS values (187.7 and 187.8 Å^2^)^48,51^ and three ^TW^CCS values (193.2, 192.2, and 191.0 Å^2^)^49,52,55^ were found for the [M + H]^+^ adduct of bisoprolol; thus, six CCS deviations were finally calculated for this ion. Among these 580 CCS deviations, 482 (83.1%) had values lower than 2%. There are five CCS deviations higher than 5%, four of which were from [M + H]^+^ ions and the fifth was from an [M + Na]^+^ ion. Factors other than the inherent difference between different types of IMS devices could also lead to large CCS deviations. For example, the maximum CCS deviation (6.2%) was found between the CCS values (177.1 and 188.1 Å^2^) of the [M + H]^+^ adduct of ciprofloxacin, published in Stephan et al.^48^ and Tejada-Casado et al.,^50^ respectively. These two CCS values are, in fact, derived from the different protomers of ciprofloxacin, as observed in the studies of Hines et al.^34^ and McCullagh et al.^62^

#### 3.2.2 CCS of Cations versus Anions

Table S3 and Figure S2 present the distribution of CCS records across different ionic species. A total of 7409 CCS values for cations, detected for 3835 compounds, were retrieved from the current publications, which represents approximately 79% of the CCS values in the compendium. The remaining 1998 CCS values of anions from 1477 compounds comprised 21% of the CCS compendium, most of which (1046) were from the FDA-approved drug profiling CCS database.^52^ In this compendium, 1142 compounds show CCS values in both positive and negative ion modes, and 335 compounds were detected only in negative ion mode (see Figure S3). The predominance of CCS data in positive ion mode was also observed in the CCS compendium of May et al.^36^ In some literature consulted for our compendium, only the CCS values of cations were determined,^33,46,47,49−51,55,56^ and these publications are associated with the detection of pesticides and drugs. This is expected from the perspective of chemical detection, as most pesticides and drugs contain carbonyl oxygen or amine in their structures, which are preferentially ionized in positive ion mode. On the other hand, some compounds are preferentially detected in negative ion mode, and these compounds include hindered phenol antioxidants, bisphenols, lubricants, PFAS, and hydroxylated PAHs, PCBs, and PBDEs, which generally contain hydroxyl or carboxyl groups in their structures.

#### 3.2.3 CCS Detected with Different Ionization Techniques

Electrospray ionization (ESI) is one of the most used ionization techniques for detecting semipolar and polar molecules. In this CCS compendium, 9005 CCS values were measured using an ESI-IMS-HRMS platform. Only approximately 4% (402) of CCS values from three publications were obtained using atmospheric pressure chemical ionization (APCI) or atmospheric pressure photoionization (APPI) methods in conjunction with an IMS-HRMS platform.^57−59^ With closer observation, we noticed that these three publications mainly worked with low-polarity compounds, such as PAHs, PCBs, and PBDEs. These compounds can form protonated or radical ions in the positive ion mode of APCI and APPI. In addition, PCBs and PBDEs can be ionized as [M – Cl + O]^−^ and [M – Br + O]^−^ species, respectively, in the negative ion mode of APCI and APPI.

A comparison of CCS values acquired from GC–APCI–TWIMS–HRMS and LC–ESI–TWIMS–HRMS was conducted by Izquierdo-Sandoval and co-workers.^58^ A high level of consistency between the CCS values measured by both platforms was observed, with 83.3% (70 out of 84) of the molecules showing CCS deviations of less than 1% and 98.8% (83 out of 84) having CCS deviations under 2%. This result indicates that the CCS values measured by either GC or LC instrumentation can be confidently used across different laboratories and instrument types.

### 3.3 CCS Data Curation and Comparison

#### 3.3.1 Consolidation of Duplicate CCS Records

The collected CCS data were subsequently unified because multiple CCS values of given ions can appear across different publications. We retrieved the chemical information for each compound from PubChem^63^ using the R package *webchem*,^64^ including the monoisotopic mass, molecular formula, canonical SMILES, isomeric SMILES, and InChIKey. The InChIKey was used as a unique identifier; the CCS values for compounds with the same InChIKey and the same ion species were unified; and the median, mean, and relative standard deviations (RSDs) of the CCS values were determined.

A total of 7017 CCS records were retained after the consolidation of duplicate records, which included 5311 CCS values of cations (3360 [M + H]^+^, 1664 [M + Na]^+^, 53 [M + NH_4_]^+^, 73 [M + H – H_2_O]^+^, 104 [M]^+*^, 32 [M + K]^+^, 9 [M + H – NH_3_]^+^, 5 [M – Na + 2H]^+^, and 11 [M – Cl]^+^) and 1706 CCS values of anions (1329 [M – H]^−^, 258 [M + HCOO]^−^, 31 [M – H – CO_2_]^−^, 26 [M – Cl + O]^−^, 22 [M + CH_3_COO]^−^, 24 [M + Cl]^−^, and 16 [M – Br + O]^−^). The distribution of CCS values across different ion species is shown in Table S3 and Figure S4. The CCS values detected for [M + H]^+^, [M + Na]^+^, and [M – H]^−^ represent approximately 90% (6353) of the CCS compendium. Some adducts were formed by the ionization of specific types of compounds; for example, [M – H – CO_2_]^−^ ions were obtained for the ionization of PFAS, and [M – Cl + O]^−^ and [M – Br + O]^−^ ions were detected for PCBs and PBDEs, respectively.

#### 3.3.2 Comparison of CCS Values from Different Publications

**Figure 3:**
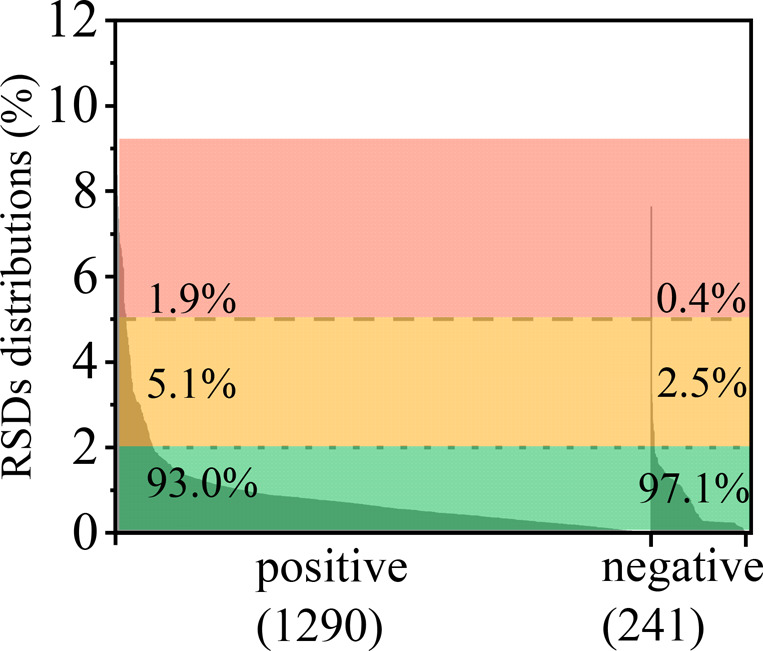
Distribution of relative standard deviations of the CCS data.

The relative standard deviations (RSDs) of CCS values were also calculated for the ions showing multiple CCS values in different publications, and the distribution of RSDs is shown in Figure 3 and Table S4. A total of 1531 ions showed multiple CCS values, including 1290 cations and 241 anions. The RSDs ranged from 0 to 10.98%, with a median value of 0.57% and a mean value of 0.84%. Generally, 6.3% (97) of ions present RSDs values higher than 2%, of which 90 ions were detected in positive ion mode, with 82 of these detected as protonated adducts. The RSDs values of anions were much lower, with more than 97% (234 out of 241) of ions showing RSDs lower than 2%, and only the [M – H]^−^ adduct of efavirenz having an RSD value (7.64%) higher than 5%. Several reasons leading to high CCS discrepancies across different laboratories were discussed in the study of Song and co-workers^35^ and include the presence of protomers, inconsistent calibration, and post-IMS dissociation of noncovalent clusters. These reasons also explain the high CCS discrepancies in this compendium; a more detailed discussion is in Supporting Information.

## 4 Machine Learning-Based CCS Predictions

The establishment of experimental CCS databases is certainly impeded by the general lack of commercially available authentic standards. Although several CCS databases, containing thousands of CCS values, have been created for environmental OMPs, many compounds remain that are not included in such databases. To enable CCS to be used in SSA and NTA for compounds that do not have experimentally measured CCS values, theoretical CCS calculated by quantum chemistry and predicted CCS generated by machine learning (ML) approaches can be used as alternatives. The quantum chemistry-based CCS calculation generally involves the procedures of ionization site prediction, conformer generation, and optimization,^65^ which can achieve 3–5% mean or median relative errors when compared against experimental CCS values.^66−69^ By contrast, predicted CCS values obtained from ML approaches have relatively higher accuracy, with mean or median relative errors ranging from 0.5% to 3%.^68,70−72^ Additionally, quantum chemistry-based methods enable the calculation of theoretical CCS values for protomers, but the calculation process may need high-performance computing resources, posing challenges to the widespread application of this method. Therefore, considering the accuracy and speed, ML approaches have greater potential for predicting the CCS values of large-scale environmental OMPs. In the following section, a selection of publicly accessible and in-house CCS prediction tools are introduced, and the factors affecting the prediction accuracy are discussed. Detailed descriptions of quantum chemistry-based CCS calculations can be found in Borges et al.^65^

### 4.1 Workflow of CCS Prediction

The fundamental workflow of CCS prediction is to use ML approaches to correlate experimental CCS values with corresponding molecular descriptors and then to use the correlation to predict the CCS values of unknowns using calculated molecular descriptors. Topological, constitutional, and electronic descriptors are commonly used for CCS prediction; more detailed introductions of these descriptors are given in the Supporting Information. Predicted CCS values are valuable and necessary due to the lack of experimental CCS values for compounds with no commercial standard. The first and most challenging step of the CCS prediction workflow using ML approaches is collecting and curating all of the experimental CCS data of interest. If the goal of the model is to achieve accurate CCS predictions for diverse compounds, then the training set of the model should contain compounds of diverse chemical classes, covering a wide chemical space. A total of 5119, 7325, 7405, and 2439 CCS records belonging to different chemical classes were used to develop AllCCS,^68^ CCSondemand,^73^ CCSbase,^74^ and DeepCCS,^72^ respectively. On the other hand, if the goal is to build a CCS prediction model for specialized compounds, then structurally similar compounds should be included in the training set. For example, 458 CCS values for lipids were used to develop a CCS prediction tool for lipid and lipid-like compounds.^75^ In this review, the CCS compendium that we have curated can serve as a training set for predicting the CCS of OMPs. After the curation of experimental CCS data, the molecular descriptors are calculated, and the data set is then split into a training set and a testing set, The training set is used for the calibration and optimization of the model, and the testing set is used for the external validation and to avoid data overfitting.

### 4.2 CCS Prediction Tools

In recent years, several comprehensive or specialized CCS prediction tools have been developed by various laboratories and groups (Table S5). The first CCS prediction tool, MetCCS, was developed by Zhou and co-workers in 2016,^76^ for which 796 CCS values of metabolites were correlated with 14 molecular descriptors, using support vector machine (SVM). Subsequently, the same research team introduced a specialized CCS prediction tool for lipids in 2017, LipidCCS,^75^ and a more accurate CCS prediction Web server for metabolites in 2020, AllCCS.^68^ However, high prediction errors were found when the AllCCS model was applied to the prediction of CCS values of mycotoxins^32^ and food contact chemicals (FCCs).^35^ For example, among the 446 compounds in the [M + H]^+^ set of FCCs, only 56.3% of predictions from AllCCS fell within 3% error, and 77.1% of predictions were within 5% error. Additionally, the CCS values of antioxidants, ultraviolet absorbers, and oligomers cannot be accurately predicted.^35^ CCSbase is another comprehensive CCS prediction tool developed by Ross and co-workers,^74^ which was also based on the SVM algorithm. The main difference between CCSbase and AllCCS lies in that CCSbase first divided the data set into several subsets by unsupervised clustering and then built CCS prediction models for each cluster. The partition allows the model to learn the specific patterns in each cluster, thus providing more accurate predicted CCS values than the model trained by all data. However, when CCSbase was applied to predict the CCS values of plastic-related chemicals, relatively large deviations were observed. Taking the results of [M + H]^+^ as an example, 47.3, 60.8, and 77.1% of predictions in the testing set showed prediction deviations within 2, 3, and 5%, respectively.^35^ A similar performance was also observed when CCSbase predicted the CCS values of mycotoxins, with 50.3% of tested mycotoxins showing prediction errors within 2%.^32^ The high discrepancies of the CCS values predicted by AllCCS and CCSbase are likely due to the structural dissimilarity between their training sets and the predicted molecules. Broeckling and co-workers^73^ also developed a comprehensive CCS prediction tool: CCSondemand, by correlating 7325 ^TW^CCS_N_2__ values with 200 descriptors via the Extreme Gradient Boosting (Xgboost) algorithm. Due to the large chemical space covered by the training set and the same type of CCS values in the model, it was observed that CCSondemand presented higher CCS prediction accuracies for plastic-related chemicals than AllCCS and CCSbase.^35,77^ DeepCCS and CCSP 2.0 are two Python-based command line tools that enable comprehensive prediction of the CCS values for small molecules. DeepCCS^72^ was trained and tested on 2439 ^DT^CCS_N_2__ and ^TW^CCS_N_2__ values using a deep neural network algorithm, which can provide a mean relative error (MRE) value of 2.7%. It has been reported that AllCCS outperforms DeepCCS in predicting the CCS values of the compounds from various super classes.^68^ CCSP 2.0 is an open-source Jupyter Notebook tool based on linear SVM, which can provide MRE values of 1.25, 1.73, and 1.87% when tested on the 170 [M – H]^−^, 155 [M + H]^+^, and 138 [M+ Na]^+^ adducts.^70^ The main advantage of DeepCCS and CCSP 2.0 is that a new CCS prediction model can be developed by using a customized training set; however, the use of a command line also presents challenges for researchers who are not familiar with Python.

Because comprehensive CCS prediction tools may provide less accurate prediction results for a specific class of molecules, some laboratories have developed specialized CCS prediction tools for compounds of interest, such as pesticides,^46^ phenolics,^78^ drugs,^49^ and plastic chemicals.^71^

Five specialized CCS prediction tools were developed for environmental OMPs.^46,49,59,71,79^ The number of CCS values, algorithms, and adducts to which they can be applied are described in Table S5. These five CCS prediction models were developed for different types of environmental contaminants, including pesticides, drugs, plastic-related chemicals, PFAS, PAHs, PCBs, and PBDEs. The number of CCS records included in four of these five models^46,49,59,79^ are less than 1000; only the work of Song et al.^71^ utilized 1721 CCS values to build the model. When considering the adducts to which these models can be applied, three only predict the CCS values of cations ([M + H]^+^ and [M + Na]^+^),^46,49,71^ the models of Celma et al.^79^ and Foster et al.^59^ further allow the prediction of CCS values of anions, such as [M – H]^−^, [M – H – CO_2_]^−^, [M – Cl + O]^−^, and [M – Br + O]^−^ adducts. It is noteworthy that in the work of Celma et al.,^79^ the CCS prediction model developed by using [M + H]^+^ data was applied to predict the CCS values of [M – H]^−^ adducts. Generally, these specialized CCS prediction tools can provide prediction errors of 5–6% for 95% of the molecules in the testing set. However, in the study of Foster and co-workers,^59^ much higher prediction accuracies were observed in two CCS prediction models developed for PBDEs/PCBs and PAHs, with all the predictions in the testing set falling within 3% and 4%, respectively. The high prediction accuracy of these two models is likely due to the high structural similarity between the compounds in the training and testing sets; it could also be related to the fact that only one type of CCS (^DT^CCS_N_2__) was included in the data set.

Currently, a comprehensive CCS prediction tool has not been developed for environmental OMPs. Song and co-workers^71^ made efforts to develop a CCS prediction tool by combining the CCS values of plastic-related chemicals and pesticides; however, this tool was only developed for cations, and the applicability of the model to other types of contaminants, such as PAHs, PCBs and PBDEs, was not evaluated. In addition, a publicly accessible CCS prediction tool is needed for environmental OMPs. Celma and co-workers^79^ have developed a publicly accessible webpage to predict the CCS values of [M – H]^−^, [M + H]^+^, and [M + Na]^+^ adducts for OMPs; this could be of great help for environmental researchers working with IMS-HRMS. However, the main drawback of this tool is that the molecular descriptors of compounds of interest need to be first calculated in OCHEM (www.ochem.eu), and then filled in the editable fields. The relatively time-consuming procedures may decrease the usability of this tool. Therefore, an easy-to-use, efficient, and publicly accessible CCS prediction tool is still needed in the environmental field.

### 4.3 Factors Affecting the CCS Prediction Accuracy

Three main factors affecting the accuracy of the predicted CCS values can be identified: the training set, the molecular descriptors, and the algorithms. Generally, the training set of the CCS prediction models should be representative of the chemical classes for the intended predictions. Zhou et al.^68^ excluded lipid-like compounds from the training set, rebuilt the model, and found that the predicted CCS values of lipid-like compounds in the testing data set had significantly larger errors after the exclusion of lipids from the training set, while the prediction results of other super classes remained similar to those of the original model. This confirmed that the chemical space of the training set contributes significantly to prediction accuracy. Currently, the training set of CCS prediction models may contain experimental CCS values gathered from different IMS devices, and the discrepancies between different instrument types can affect the accuracy of the model. The curation of CCS values from different instrument platforms into a single database can provide high chemical diversity but also has the potential to introduce variations; therefore, the suitability of combining different types of measured CCS values in the model needs to be evaluated. The study of Song and co-workers^71^ developed a CCS prediction model by using both ^DT^CCS_N_2__ and ^TW^CCS_N_2__ values; when the researchers excluded the CCS values of organophosphate flame retardants and phthalate monoesters detected using a DTIMS device from the training set, the predicted CCS values for the [M + Na]^+^ adducts of similar compounds were less accurate. Thus, in some cases, the use of CCS values from different IMS types for model development is required as the experimental CCS values of some compounds have only been measured by a single type of IMS, and these CCS values are indispensable for building the prediction model.

Recently, Richardson and co-workers proposed an improved CCS calibration approach for TWIMS systems,^80^ which has the potential to further improve the consistency between experimental ^TW^CCS_N_2__ and ^DT^CCS_N_2__ values, and thereby lead to more accurate CCS prediction models.

The molecular descriptors used in the prediction models can also affect the prediction accuracy. Currently, the molecular descriptors used in the models are mainly calculated from neutral molecules. However, measured CCS values are based on the mobility of ionized molecules, such as [M + H]^+^ and [M + Na]^+^ ions. This discrepancy will inevitably introduce prediction errors. A sodium cationized ion has a higher atomic radius compared to a protonated ion,^29^ and therefore the conformational difference between sodium adducts and neutral molecules may be larger than the difference between protonated adducts and neutral molecules. This goes some way to explaining why the predicted CCS values of sodium adducts always show larger errors than the predicted CCS values of protonated adducts.^46,77^ Several approaches for predicting the CCS values of multiple adducts have been adopted in the current CCS prediction models. The first approach is to integrate all the CCS data of multiple adducts into one data set and then use this data set to develop one model to simultaneously predict the CCS values of different adducts. It is important to note that the adduct information must be incorporated into the final data set. This method was widely utilized in the current CCS prediction tools, such as LipidCCS,^75^ MetCCS,^76^ AllCCS,^68^ and CCSbase.^74^ Taking MetCCS as an example, 14 descriptors from the Human Metabolome Database (HMDB) were used to develop the CCS prediction model for metabolites; in these 14 descriptors, the actual *m*/*z* of the ion instead of the accurate mass of neutral molecules is used for prediction. The study of Ross and co-workers^74^ employed another method to incorporate the adduct information in the data set, in which the MS adduct was converted into a binary representation using one-hot encoding. The main advantages of integrating multiple adducts into one data set are that only one CCS prediction needs to be developed and the CCS values of multiple adducts can be simultaneously predicted. However, this approach may lead to low prediction accuracy for some adducts, as a different set of descriptors is needed when predicting the CCS values of different adducts.^71,79^ The second approach to predicting the CCS values of different adducts involves developing a unique prediction tool for each adduct. This approach was adopted in the studies of Rainey et al.,^70^ Celma et al.^79^ and Song et al.^71^ and has the potential to provide more accurate prediction results for each adduct. However, more training and optimization are required when developing the CCS prediction model, and it is not applicable to ion species with few CCS records.

Some works have studied the effect of algorithms on the accuracy of the CCS predictions. In the work of Gonzales and co-workers,^78^ three algorithms, stepwise multiple linear regression (SMLR), principal components regression (PCR), and partial least-squares regression (PLS), were used to develop CCS prediction models. The results showed that PCR and PLS provided more accurate predictions than SMLR. In the study of Song et al.,^77^ SVM was found to be able to provide more accurate predictions than PLS. In addition, Ross et al.^74^ found that SVM also outperformed least absolute shrinkage and selection operator (Lasso) and random forest (RF) regression. SVM was used for the development of AllCCS, CCSbase, MetCCS, and LipidCCS (Table S5). The wide application of SVM is due to its easy configuration with few hyperparameters as well as its ability to provide accurate and reproducible prediction results.

Currently, the CCS values of [M + H]^+^, [M + Na]^+^, and [M – H]^−^ species are more accurately predicted by ML approaches than the CCS values for other adducts; this is partly due to the large number of experimental CCS values published for these three ion species. For example, the CCS values of [M + H]^+^, [M + Na]^+^, and [M – H]^−^ species represent approximately 91% of the compendium assembled here. Another reason for the more accurate predictions for these three adducts is that most CCS prediction tools are built using the descriptors of neutral molecules, and ions with a larger atomic radius are more likely to cause larger conformational changes to molecules. Therefore, it is challenging to accurately predict the CCS of ion species with large atomic radii by using molecular descriptors from neutral molecules.

Currently the deviation of most of the predicted CCS values from measured values can be as high as 6%. In the study by Bijlsma et al.,^46^ approximately 95% of predicted CCS values of protonated molecules had prediction errors of less than 6%. In the study of Song and co-workers,^71^ more than 93% of protonated molecules and 95% of sodiated molecules showed prediction errors of less than 5%. The study of Rainey et al.^70^ showed similar prediction performance, in which approximately 92%, 90%, and 92% of compounds in [M + H]^+^, [M + Na]^+^, and [M – H]^−^ testing sets, respectively, had prediction errors within 5%. This prediction error is still too large to be confidently used for isomer differentiation, as many isomeric pairs have CCS differences of less than 5%.^81^ Consequently, predicted CCS values are mainly used to help eliminate false positives and improve the confidence level of identification in SSA.

## 5 Use of IMS and the Derived CCS for the Analysis of OMPs

With the rapid growth of published CCS data and the increasing resolving power (*R*_p_) of commercial IMS techniques, IMS in combination with GC– or LC–HRMS systems is increasingly applied to the analysis of small molecules, such as metabolites, phenolic compounds, and food contaminants.^82−84^ As CCS is related to the shape and size of ionized molecules, distinct CCS versus *m*/*z* trend lines have been observed for the different classes of compounds. In this CCS compendium, an average CCS value of 195 ± 52 Å^2^ was observed for compounds with a molecular weight of approximately 400 Da (see Figure 1A). This level of CCS variation is enough to separate some coeluting analytes, thereby reducing the false positives and facilitating the identification of unknown compounds. The information provided by CCS, which is complementary to *m*/*z* and RT, is especially helpful in the analysis of complex environmental samples. Celma et al.^21^ have established five confidence levels for the identification of compounds by applying IMS-HRMS instruments; this five-level criterion was based on the matching of *m*/*z*, retention time, CCS, and MS/MS spectra. Compared to the identification levels proposed by Schymanski and co-workers,^85^ the addition of CCS matching has the potential to filter out some isomeric candidates and improve the identifications from level 3 (tentative candidates) to level 2 (probable structure). In this section, we summarize the application of IMS and the derived CCS to the analysis of different types of OMPs, including pesticides, pharmaceuticals, PFAS, PAHs, PCBs, PBDEs, plastic-related chemicals, mycotoxins, and steroids.

### 5.1 Pesticides and Pharmaceuticals

Pesticides and pharmaceuticals are two main types of widely distributed environmental OMPs, which can originate from agricultural runoff and hospital effluent, respectively.^86,87^ Since pesticides and pharmaceuticals are usually simultaneously monitored or screened in environmental samples, the application of IMS to the analysis of these two types of contaminants is discussed here.

Some research groups have incorporated IMS into their GC– or LC–MS systems with the aim of removing interfering ions and improving the selectivity of the analytical methods. Celma et al.^88^ combined LC–IMS–QTOF with targeted analysis and SSA to monitor the OMPs in coastal lagoons and estuaries across the Spanish Mediterranean coastline. A total of 96 OMPs were identified in surface water samples, with pesticides and pharmaceuticals being the most frequently detected chemicals. The study of Hinnenkamp and co-workers also combined the LC–IMS–QTOF with targeted analysis, SSA, and NTA to comprehensively characterize the contaminants from wastewater treatment plant effluent to drinking water. A total of 104 compounds were unequivocally or tentatively identified, with most of them originating from pharmaceuticals and transformation products.^89^

In the targeted analysis and SSA, the general criteria used to match a detected feature to a target compound are RT deviations < 0.1 min, *m*/*z* error < 5 ppm, and at least one fragment ion to be found.^88,90,91^ However, many pesticides are in such low abundance that there is insufficient ion intensity for the formation of fragment ions; therefore, true identifications may be discarded by applying these criteria. Regueiro et al.^92^ spiked 156 pesticides with fish feed samples at different levels (0.01, 0.05, 0.20 mg/kg) and compared the detection rates by applying different screening criteria. The results showed that at 0.01 mg/kg, the addition of the fragment criterion to the *m*/*z* and RT filters dramatically decreased the detection rate from 70.4% to 42.4%. For the identification of pesticides at trace levels, the combination of CCS in conjunction with *m*/*z* and RT criteria could be an ideal choice, since the addition of a CCS filter (±2%) showed negligible effect on the detection rates, while significantly reducing the number of false positives.^92^

CCS is also helpful for discovering the metabolites and transformation products (TPs) of pesticides and pharmaceuticals. Bijlsma et al.^93^ investigated the metabolites of the insecticide pirimiphos-methyl through SSA. The predicted RT and CCS values can narrow down the candidate list (38–66% reduced) and five metabolites of pirimiphos-methyl were tentatively identified, two of which were further confirmed with reference standards. Besides the SSA, NTA has also been used to identify metabolites or TPs. In the study by Hinnenkamp and co-workers,^89^ metoprolol acid/atenolol acid was identified as a TP of metoprolol or atenolol, and 1,3-benzothiazol-2-sulfonic acid was identified as a TP of 2-mercaptobenzothiazole. In all of these cases, the identifications of TPs are mainly based on mass spectra, but they are also supported by CCS measurements. The use of CCS data in the identification of metabolites and TPs is still in an early stage as many of these “new” compounds do not have experimental CCS values due to the lack of commercial standards. Additionally, predicted CCS values do not yet provide sufficient accuracy to enable isomer differentiation.

### 5.2 PFAS

PFAS are a large group of synthetic chemicals containing a chain of linked carbon and fluorine atoms, which are widely used in consumer and industrial products.^94,95^ Due to the high stability of the C–F bond, PFAS do not degrade easily in the environment, which leads to their environmental persistence and high bioaccumulation potential.^96^ Currently, more than 14 000 unique PFAS are listed in the CompTox Chemicals Dashboard of the United States Environmental Protection Agency (EPA),^97^ and this number is constantly increasing with the advancement of detection technologies and data processing methods.

The combination of CCS with RT and *m*/*z*, together with other tools, such as mass defect and homologous series evaluation, can provide higher confidence in assigning unknown PFAS structures. Luo et al.^98^ utilized LC–IMS–QTOF coupled with NTA to characterize the PFAS in aqueous film-forming foams. Thirteen known PFAS and 20 new PFAS-like homologous series were discovered following a feature prioritization process employing *m*/*z*, CCS, mass defect matching, homologous series search, and MS/MS fragmentation experiments. The study by Valdiviezo et al.^99^ used an untargeted LC–IMS–QTOF analysis approach and discovered 26 PFAS in the surface water of Houston Ship Channel/Galveston Bay.

A large number of isomers have been found for PFAS, which can result from the different production processes and transformation pathways.^100^ Given that different isomers can lead to different environmental behavior and biological effects,^101^ the unequivocal identification of isomers of PFAS is vital for evaluating potential health risks. Several studies^22,102−104^ have investigated the separation of linear and branched isomers of PFAS, such as perfluorooctanoic acid (PFOA), perfluorooctane sulfonic acid (PFOS), and perfluorohexane sulfonic acid (PFHxS), using both IMS and LC separation. Generally, dimethylated isomers possess the most compact structures and have drift times lower than those for monomethylated isomers and the linear form. The complementary separation of PFAS compounds is achieved using a combination of LC and IMS analysis.^22^ In addition to the identification of isomers of PFAS, some studies have also investigated their distinct distributions in the environment. Mu et al.^104^ compared the environmental behavior of linear and branched PFAS in municipal wastewater treatment plants using a LC–IMS–QTOF platform. Linear PFAS were detected more frequently than branched isomers in wastewater samples. Additionally, the concentrations of branched PFAS were higher in effluents than in influents.

Numerous studies showed that exposure to PFAS can lead to adverse health effects at the level of μg/L or less;^96,105^ therefore, sensitive chemical analytical methods for PFAS are needed to support their further environmental fate and toxicity effect studies. The incorporation of IMS into a LC–MS/MS system can increase the S/N ratios of analytes by removing the background interferences; thus, lower limits of detection (LODs) can be obtained for the analytes.^106,107^ Gonzalez de Vega et al.^102^ utilized UPLC–TWIMS–QTOF to quantify the PFAS in Cooks River water and achieved LODs and limits of quantification (LOQs) of 0.19–0.76 μg/L and 0.56–2.30 μg/L, respectively. IMS filtering can also provide more accurate quantification results by removing the interference of coeluting compounds. Díaz-Galiano et al.^107^ showed that the peak area of perfluorodecanoic acid (PFDA) in one mussel sample was reduced by 13% after the removal of a coeluting peak. This is rather important when the concentrations of analytes need to be compared to the maximum limits established by regulatory authorities.

### 5.3 PAHs, PCBs, PBDEs, and Their Metabolites

PAHs, PCBs, and PBDEs belong to a class of compounds known as persistent organic pollutants (POPs). These compounds are of interest due to their persistence in the environment, long-range transportability, and adverse effects on human health.^108^ In addition to the parent compounds, PAHs, PCBs, and PBDEs can also form hydroxylated and methoxylated metabolites in the environment. Several studies have shown that hydroxylated PCBs or PBDEs possess higher toxicity than the corresponding parent compounds.^109−111^

Currently, only a few studies use the IMS technique to analyze PAHs, PCBs, and PBDEs. Sun et al.^112^ developed a method by coupling fabric phase sorptive extraction with IMS to detect the PAHs in aquatic environments. The study of Olanrewaju and co-workers^113^ utilized GC–TIMS–QTOF to characterize the composition of crude oil. Ma et al.^114^ adopted UPLC–IMS–QTOF to analyze hydroxylated PBDEs, and the peak capacity was increased by approximately two times after the addition of the IMS dimension.

Some studies have shown that positional isomers of PAHs, PCBs, and PBDEs would show distinct adverse health effects;^115,116^ therefore, the structural elucidation of these isomers is important in order to further understand their mechanisms of toxicity. The study of Zheng et al.^57^ showed that some isomers can be separated based on their varying collisions with buffer gas in an IMS cell, such as PCB 103 and PCB 126, PBDE 85 and PBDE 116. Adams and co-workers utilized TIMS to separate hydroxylated metabolites of PCBs and PBDEs, with a mobility resolution of at least 150 required to separate some isomeric metabolites.^106,117^ Castellanos et al.^118^ also observed that some PAH geometric isomers can be separated if mobility resolution is above 150.

To the best of our knowledge, there are no publications that use the IMS technique for the quantitative analysis of PAHs, PCBs, or PBDEs in environmental samples. However, increases of S/N ratios have been observed for hydroxylated PBDE after the removal of interfering ions by drift time alignment.^114^ The improvement in the quantitative analysis of PAHs, PCBs, and PBDEs brought by IMS needs to be further investigated.

### 5.4 Plastic Additives and Non-Intentionally Added Substances (NIAS)

Ubiquitous plastic waste has resulted in a wide distribution of plastic additives and their TPs in aquatic and terrestrial environments.^119−121^ More than 10,000 chemical substances, including monomers and additives, can be used in plastic production.^122^ In addition, non-intentionally added substances (NIAS) can also be formed in plastics due to the degradation of additives and polymers, contamination from the manufacturing process, and shelf life.^123,124^ The complexity of plastic matrices makes the full characterization of chemical components very difficult.

Several studies^24−27,60,71,77,125,126^ have used LC–IMS–MS/MS platforms to characterize plastic-related chemicals in consumer products and environmental samples. Vera et al.^27^ combined LC–IMS–QTOF with NTA to identify the nonvolatile substances migrating from polyethylene films used as food packaging. A total of 35 compounds were identified, 17 of which were NIAS. Song et al.^125^ used LC–IMS–QTOF, together with RT and CCS prediction tools, to develop a workflow for the identification of nonvolatile compounds migrating from plastic food contact materials; the authors stated that the use of predicted RT and CCS values can reduce the number of false positives in SSA. Wrona and co-workers used a LC–IMS–QTOF platform to study dishes made from biomaterials.^127^ They discovered plasticizers, lubricants, and oligomers in the dishes that most likely originated from the adhesives used in the manufacture of the bio-based dishes.

Analyses of plastic-related chemicals in environmental samples have also been undertaken in some studies.^60,71^ In the study of Song and co-workers,^71^ a LC–IMS–QTOF-based SSA approach was used to identify the plastic-related chemicals in Ebro river water and a total of 98 compounds were tentatively identified including both plastic additives and NIAS. Organophosphorus flame retardants were also detected in indoor dust in the work of Mullin et al.^60^

Isomers of plastic-related chemicals have also been separated and identified based on their distinct CCS values, for example, the positional isomers of flame retardants tri-m-tolyl phosphate ([M + H]^+^ 188.6 Å^2^, [M + Na]^+^ 198.6 Å^2^), tri-*o*-tolyl phosphate ([M + H]^+^ 182.4 Å^2^, [M + Na]^+^ 192.4 Å^2^), and tri-*p*-tolyl phosphate ([M + H]^+^ 190.0 Å^2^, [M + Na]^+^ 200.0 Å^2^).^20^ In some cases, however, the difference in the CCS values of isomers is less than 2%, which is too small to be resolved by current IMS instrumentation. Examples of such isomers include tributyl phosphate ([M + H]^+^ 166.7 Å^2^) and tri-isobutyl phosphate ([M + H]^+^ 165.4 Å^2^),^20^ and di-isoalkyl phthalates and dialkyl phthalates.^35^ To definitively identify such isomers would require an IMS device with higher *R*_p_ and better reproducibility ultimately providing CCS measurements reproducible to within 0.5%.^128^

### 5.5 Mycotoxins and Steroids

Mycotoxins and steroids are two types of environmental OMPs that have received increasing attention in recent years. Mycotoxins are toxic secondary metabolites produced by various mold species and are commonly detected in foods and feeds. In recent years, the presence of mycotoxins in indoor dusts has been verified in some studies.^129,130^ Steroids, both endogenous and exogenous synthetic ones, play important roles in biochemical and physiological processes. Some steroids can be used as human and veterinary drugs or doping agents in sports, and the increased use of steroids results in them being widely distributed in aquatic environments.^131^

A few studies have employed IMS techniques to analyze mycotoxins and steroids in food or environmental samples,^23,132,133^ Fan et al.^23^ utilized a LC–IMS–QTOF platform to determine the presence of 20 mycotoxins in 130 fruit samples. In addition to the analysis of the parent mycotoxins and steroids, studies incorporating CCS data were performed to identify their metabolites. Hernandez-Mesa et al.^132^ incorporated CCS into the identification process of steroid metabolites. One metabolite of boldione was successfully identified as 17α-boldenone glucuronide rather than 17β-boldenone glucuronide due to the different CCS values of their protonated adducts. Righetti et al.^133^ used theoretical CCS values in order to differentiate isomers of mycotoxin glucuronide metabolites. However, the deviations between theoretical and experimental CCS values ranged from 0.7% to 8.8% and were too large to confidently differentiate isomers.

Some isomers of steroids can be directly separated by IMS and identified by their distinct CCS values, such as protonated pregnenolone and 5α-dihydroprogesterone, with CCS values of 176.7 Å^2^ and 191.4 Å^2^, respectively.^134^ However, in most cases, the steroid isomers possess highly similar structures and cannot be separated by current IMS techniques. Other techniques to differentiate steroid isomers have also been investigated, including derivatization of steroids prior to IMS analysis, using multimeric ion species, and changing the drift gas environments. Velosa et al.^135^ employed a derivatization strategy using 1,1-carbonyldiimidazole (CDI) to target the C17 hydroxyl group of endogenous steroids, an effective increase in IMS resolution of more than 15% was observed for some derivatized stereoisomers. Similarly, an improved separation efficiency for steroid isomers was observed following derivatization by *p*-toluenesulfonyl isocyanate.^136^ In addition to the derivatization method, Chouinard et al.^134^ and Rister et al.^137−139^ separated and identified isomeric steroids using multimeric metal adducts. In the study by Chouinard and co-workers,^134^ testosterone and epitestosterone were confidently differentiated via a large CCS variation (3.5%) of their [2M + Na]^+^ adducts. Other multimeric ion species, such as [2M + Li]^+^, [2M + K]^+^, and [3M + K]^+^, were also used to differentiate steroid isomers.^138,139^ Changing the drift gas is another method used to improve the mobility separation of steroid isomers. In the study by Chouinard et al.,^134^ using CO_2_ as the drift gas instead of N_2_, provided better mobility separation for the sodiated dimer of epitestosterone and dehydroepiandrosterone. However, since most of the current published CCS values are measured using N_2_ as the drift gas, universally changing to CO_2_ is unlikely for the routine analysis of steroid isomers.

As in the case of PFAS, improvement of quantitative analysis on incorporation of IMS was also observed for the analysis of steroids. The elimination of interference from coeluting matrix compounds and background noise improves the S/N ratios of analytes. In the study by Hernández-Mesa et al.^132^ the S/N ratios were improved 2–7-fold after the addition of TWIMS filtering, a similar improvement in S/N ratios was also observed for DMS filtering.^140^ However, the addition of IMS to LC–MS/MS systems does not always improve the S/N ratios. In the study by Lindemann et al.,^141^ improvements in LODs were observed for 21 of 34 mycotoxins after the addition of TIMS separation. This was mainly due to the lower accumulation times resulting from the strong matrix load introduced into the TIMS cartridge. Fortunately, technological development is ongoing, and a parallel accumulation mode of TIMS that enables ion accumulation and analysis to occur together has the potential to achieve a 100% duty cycle.^142^ The TIMS operated in parallel accumulation mode can provide the advantage of high selectivity without losing the sensitivity.

## 6 Benefits of IMS and the Derived CCS in Targeted, Suspect, and Non-Targeted Screening Analysis of OMPs

Through the summary of the use of IMS and the derived CCS in the analysis of OMPs, we found that the addition of IMS into GC– or LC–MS can improve the selectivity of the method, decrease the LODs of analytes, and bring some benefits for targeted analysis, SSA, and NTA. In this section, four main advantages brought by the IMS technique are discussed, including increasing peak capacity, elimination of interference, separation of isomers, and finally the reduction of false positives and false negatives.

### 6.1 Increasing Peak Capacity

The first advantage of IMS is increasing the peak capacity of conventional LC–MS/MS by adding another separation dimension.^41,143^ IMS can separate ionized molecules based on their size, shape, and charge, which provides complementary molecular information for compounds in addition to conventional RT and *m*/*z*. Compounds with different structural characteristics tend to exhibit distinct relationships between *m*/*z* and CCS values, and this has been verified by the different CCS versus *m*/*z* trend lines of plasticizers, OPFRs, and PFAS.^20^ A similar phenomenon was also observed in other publications.^22,34,35,144^ Generally, the mass–mobility relationship is affected by the elemental composition and molecular structure of the compounds. On comparing compounds with the elements C, H, and O and alkyl groups to those with halogens and aryl groups, the latter group will generally have smaller CCS values for a given *m*/*z*. The studies of Haynes et al.^145^ and Arthur et al.^146^ showed that the implementation of IMS in LC–MS workflows increased peak capacity at least 2–3-fold, due to the elimination of chemical noise and separation of coeluting isobaric species. Improving the *R*_p_ of IMS devices would further increase the peak capacity of LC–IMS–MS systems.

### 6.2 Elimination of Interference by Drift Time Alignment

**Figure 4:**
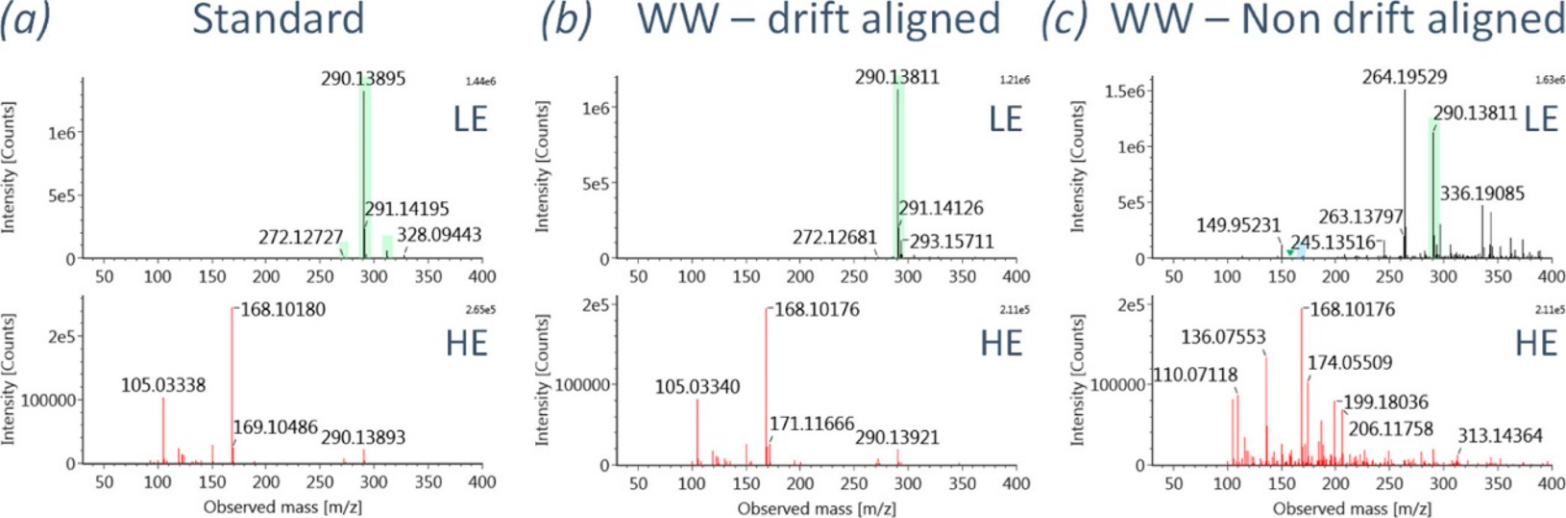
Comparison of HRMS spectra for benzoylecgonine from an analytical reference standard solution (a), DT-aligned data of positive finding in wastewater sample (b), and non-DT aligned data of the same positive finding in wastewater (c). Reprinted with permission from ref (21). Copyright 2020 American Chemical Society.

Another advantage of IMS is that it can eliminate interference from coeluting compounds and background noise, which can provide “cleaner” mass spectra and lower LODs.^21,140^ When IMS separation occurs before precursor ion fragmentation in IMS–HRMS platforms, the precursor ion and its corresponding product ions share the same drift time. The alignment of precursor and fragment ions based on both RT and drift time can eliminate many of the interfering ions from coeluting compounds or background noise, simplifying the mass spectra and subsequent spectral interpretation. This advantage of IMS has been shown in many research articles.^21,24,33,90^Figure 4 shows the mass spectra of benzoylecgonine in wastewater samples with and without drift time alignment. It can be seen that the most abundant ion with *m*/*z* of 264.1953 and other interfering ions were removed following drift time alignment. The drift-time-aligned mass spectra (Figure 4b) are easier to interpret and are more comparable to the mass spectra of the corresponding reference standard.

The elimination of background interference can also lead to an increased signal-to-noise (S/N) ratio; thus lower LODs for analytes of interest can be obtained,^140,147,148^ The study of Carbonell-Rozas and co-workers showed that the integration of TWIMS in the LC–MS/MS workflow improves the S/N between 2.5 and 4 times.^149^ The decrease in LODs makes it possible to detect contaminants at lower concentrations in environmental samples.

### 6.3 Isomer Separation and Identification

Isomer separation and identification is another attractive benefit of using the IMS technique in environmental analysis.^118,150^ As isomeric species have the same molecular mass and often share similar fragmentation patterns, they are often indistinguishable from MS alone. Even if the isomers can be chromatographically separated, further confirmation still requires authentic standards. The different spatial conformations of isomers provide the possibility for their separation and identification by IMS. Currently, commercial IMS systems provide interlaboratory and interplatform CCS reproducibility of around 2%;^30,32^ therefore, in theory, isomers can be separated and identified as long as the difference between their CCS values is greater than 2%. The use of IMS to differentiate between isomers of biomolecules, such as glycans, lipids, peptides, and proteins, has been previously reviewed by Wu and co-workers.^151^ Herein, we mainly focus on the isomer differentiation of environmental OMPs.

The topic of isomer differentiation is of great interest in environmental analysis, as different isomers could show different environmental behavior and biological effects.^152,153^ Several isomeric pairs of environmental OMPs have already been separated by IMS and identified by their different CCS values, as discussed in section 5. More isomeric pairs and their CCS values are shown in Table S6.

**Figure 5:**
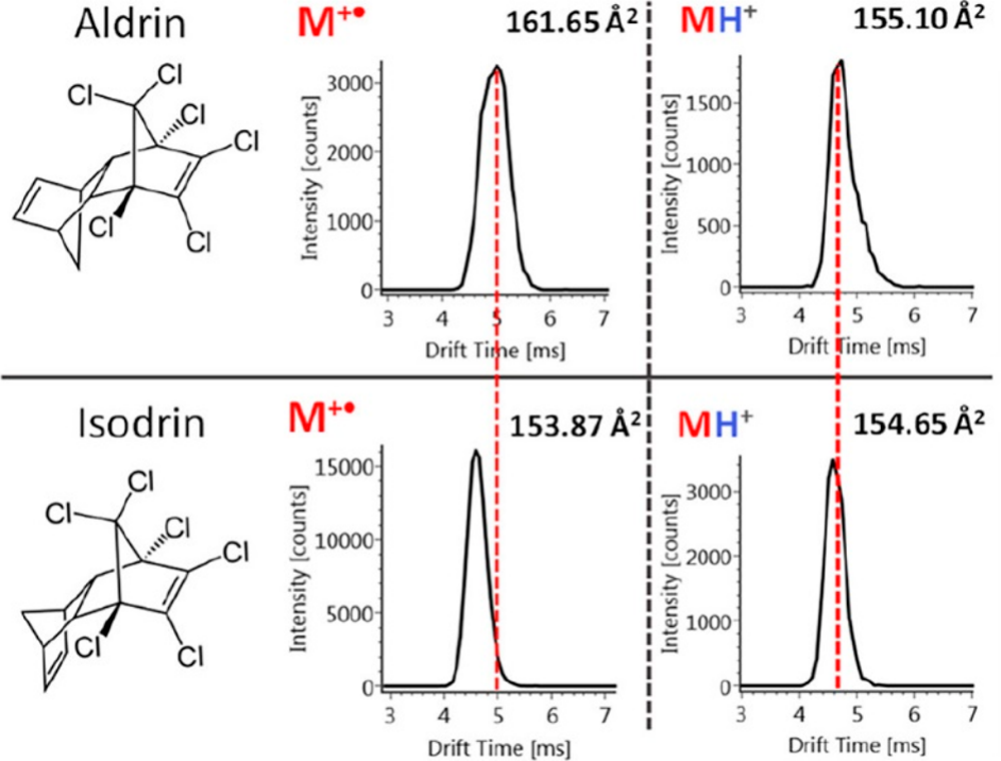
Arrival time distributions of the regioisomers aldrin and isodrin in charge transfer conditions (left) and proton transfer conditions (right). Reprinted with permission from ref (58). Copyright 2022 American Chemical Society (licensed under CC-BY 4.0, https://creativecommons.org/licenses/by/4.0/).

Sometimes, isomers can only be separated and identified according to the CCS values of one specific adduct, for example, the ergot alkaloids and their corresponding epimers can be distinguished by the CCS values of their [M + Na]^+^ adduct, but not from the CCS values of their [M + H]^+^ adduct.^149^Figure 5 shows the arrival time distributions of aldrin and isodrin; it is obvious to see that similar CCS values were obtained for their [M + H]^+^ adducts, however, the CCS values of M^+^* ions were significantly different, enabling their unequivocal identification. To aid the identification of isomers in the future, the comprehensive measurement and reporting of CCS values are necessary, since currently the adduct of isomer pairs that could show distinct CCS values enabling unambiguous identification is generally unknown.

It should be noted that although CCS is a promising parameter for isomer differentiation, variations in RT cannot be ignored. Some isomer pairs with similar CCS values have been successfully separated by their RT values.^91,104,137^ Just as stated by Fabregat-Safont et al.,^91^ the combination of multidimensional structural information, including RT, CCS, *m*/*z*, and fragments, acquired from LC–IMS–HRMS instrumentation, can enable more isomers to be separated and accurately identified.

### 6.4 Reducing False Positive and False Negative Detections

CCS values can also be used to reduce the number of false positive identifications in SSA since it provides additional identification evidence. The assignment of unknowns in current SSA mainly relies on the matching of *m*/*z* and isotopic patterns, which can result in false positive identifications. Given that the CCS is not completely correlated with *m*/*z*, the incorporation of CCS data in SSA has the potential to filter out false positive identifications and decrease the burden of manual verification. The work of Celma and co-workers has shown that the inclusion of CCS (with a tolerance of 2%) into the filtering process of pesticides and pharmaceuticals can reduce the number of false positives by 13.5–44.4%, while not affecting the true identification results.^90^ Predicted CCS values play a role similar to experimental CCS values in reducing false positives. Bijlsma and co-workers showed that using predicted CCS values, with a tolerance of 6%, reduced the number of false positives by 5%–39%.^46^ A similar reduction in false positives has been reported in other publications employing different CCS prediction tools.^70,71,125,154^

The number of false positives eliminated by applying a CCS filter is dependent on the CCS tolerance used. A smaller CCS tolerance can eliminate more false positive candidates but also increase the risk of filtering out correct identifications. Currently, a tolerance of 2% is usual for comparing experimentally derived CCS values to measured data in SSA and NTA.^30^ In the case of predicted CCS values, tolerances of 5–6% are used given the current accuracy of predicted CCS values.^46,71,93^

The reduction of false positives can be also achieved by separating the targets from the interference of coeluting compounds or background noise. In the work of Chen and co-workers,^155^ the peak of sulfotep ([M + H]^+^, *m*/*z* 323.0308) overlapped greatly with the background ions with *m*/*z* 323.0525; thus the *m*/*z* value of protonated sulfotep was recorded as 323.0372. The calculation of compound compositions based on *m*/*z* 323.0372 could lead to false identification for this peak. Simultaneously, this case also showed that the addition of IMS can avoid some false negatives since this peak cannot be identified as sulfotep without the elimination of background noise by IMS. The reduction of false negative results from IMS was also observed in other studies; in the work of Olanrewaju et al.,^113^ the chromatographic coelution of two compounds, chrysene and triphenylene, was observed; however, the peaks of these two compounds can be successfully resolved by IMS and were further identified by mass spectra and CCS values.

## 7 Current Limitations and Future Prospects

IMS techniques are being increasingly used in the analysis of environmental OMPs; however, a harmonized and standardized method for CCS measurements is still lacking. The accuracy of the current CCS measurements can affect the confidence with which they can be incorporated into screening analyses. Although an error threshold of ±2% is considered an acceptable criterion for the use of CCS databases,^21,28,92^ large deviations in the CCS values of some molecules have been observed when measured on different platforms and by different laboratories. Deviations in CCS values of up to 7% have also been observed between ^DT^CCS_N_2__ and ^TW^CCS_N_2__ values, as well as between ^DT^CCS_N_2__ and ^TIM^CCS_N_2__ values.^31,156^ The high CCS deviations for a small fraction of molecules can introduce uncertainties for the use of CCS across different laboratories and instrumental types.

The 9407 CCS values compiled in this work can be of great help for the screening analysis of OMPs in environmental samples. Simultaneously, this up-to-date CCS compendium will also benefit the development of ML-based CCS prediction tools for environmental OMPs, since the collection of experimental data is a time-consuming procedure in ML workflows. Some approaches can be used to further improve the chemical diversity of this CCS compendium, such as measuring the CCS values of multiple adducts of molecules and disclosing all of the CCS measurements under a public license. Furthermore, we also encourage researchers to incorporate the compound identifiers, such as PubChem CID, SMILES, and InChIKey, in the published CCS data. Besides, it is recommended to use the compound’s full name instead of its abbreviation when publishing the CCS data, as the latter may lead to potential misunderstandings for researchers and also bring challenges for the online retrieval of compound structural information.

ML-based CCS prediction has great potential to simplify SSA workflows due to its ability to provide predicted CCS values for compounds for which no authentic standard is available. However, there is still work to be done to develop more accurate models for environmental OMPs. Compared with algorithms and descriptors used in the models, the chemical space and quality of training data seem to be the most significant factors affecting the CCS prediction.^71^ Therefore, the models could be improved by having a more comprehensive range of high-quality CCS measurements. The comprehensiveness of the model can be achieved by collecting the CCS data from different sources; however, the quality of CCS measurements can be affected by different instrument platforms and different laboratory conditions, as pointed out in section 4.3. Thus, the suitability of combining CCS measurements from different sources and using them as a training set for a model should always be carefully evaluated. To ensure high-quality CCS measurements in the training set, using only ^DT^CCS_N_2__ data, measured by the stepped field method, to train the model is a reliable approach; however, current ^DT^CCS_N_2__ data are more extensively measured by the single field method because DTIMS operated in single field mode shows compatibility with prior chromatographic separations. The accuracy of single field ^DT^CCS_N_2__ values and ^TW^CCS_N_2__ values can be enhanced by implementing an improved CCS calibration approach. Studies into this area are currently being undertaken by research teams.^80,157^ The incorporation of such high-quality CCS measurements in the training set has the potential to further improve the CCS prediction accuracy.

The advantages of IMS in HRMS-based targeted analysis, SSA, and NTA make it a promising tool applicable to the monitoring of environmental OMPs. There remain some existing challenges in the analysis of environmental contaminants, including complex sample matrices, the presence of isomers, and low concentrations of analytes in samples, and these can be partially addressed by the addition of IMS separation. With the improvement of IMS *R*_p_, enhancements of CCS databases, and the development of more accurate CCS prediction tools, the practicability of IMS–MS in the analysis of environmental OMPs will continue to improve.

## Data Availability

Collected empirical CCS values before and after consolidation have been deposited online at 10.5281/zenodo.8375655.

## Supporting Information Available

Distribution of CCS across ion species; compounds detected in positive and negative ion modes; distribution of unified CCS across ion species; distribution of 4170 compounds across super classes; comparison between the ^DT^CCS_N_2__ and ^TW^CCS_N_2__; RSDs of repeated CCS measurements; published CCS prediction tools; isomeric pairs and their CCS values; introduction of DTIMS, TWIMS and TIMS; sources of high CCS deviations; commonly used molecular descriptors for CCS prediction (PDF)
Collected empirical CCS values before and after consolidation (XLSX)
