# Supplementary material for: Application of Ion Mobility Spectrometry and the Derived Collision Cross Section in the Analysis of Environmental Organic Micropollutants
Source: Environ Sci Technol. 2023 Dec 13;57(51):21485–502. doi: 10.1021/acs.est.3c03686 (PMC10753811; doi:10.1021/acs.est.3c03686)
Supplement: Supplementary file 1 — es3c03686_si_001.pdf [file es3c03686_si_001.pdf]

Supporting Information

Application of Ion Mobility Spectrometry and the Derived Collision Cross Section in the Analysis of Environmental Organic Micropollutants

Xue-Chao Song[1,2,3], Elena Canellas[3], Nicola Dreolin[4], Jeff Goshawk[4], Meilin Lv[2,5], Guangbo Qu[1,2,6*], Cristina Nerin[3,*], Guibin Jiang[1,2,6]

1. School of the Environment, Hangzhou Institute for Advanced Study, University of the Chinese Academy of Sciences, Hangzhou 310024, China.

2. State Key Laboratory of Environmental Chemistry and Ecotoxicology, Research Center for Eco-Environmental Sciences, Chinese Academy of Sciences, Beijing 100085, China.

3. Department of Analytical Chemistry, Aragon Institute of Engineering Research I3A, EINA, University of Zaragoza, Maria de Luna 3, 50018, Zaragoza, Spain.

4. Waters Corporation, Stamford Avenue, Altrincham Road, SK9 4AX, Wilmslow, United Kingdom.

5. Research Center for Analytical Sciences, Department of Chemistry, College of Sciences, Northeastern University, 110819, Shenyang, China.

6. Institute of Environment and Health, Jianghan University, Wuhan 430056, China

*Corresponding author:
Cristina Nerin, Phone: +34 976761873; Email: cnerin@unizar.es
Guangbo Qu, Phone: +8610 62849129; Email: gbqu@rcees.ac.cn

Total Number of Pages: 20
Total Number of Supplementary Figures: 4
Total Number of Supplementary Tables: 7

Table of Contents:

Figure S1. Distributions of the yearly publication of CCS records and the contributions of the main research groups. Different color in the same year indicate the contributions of different groups.

Figure S2. Distribution of 9407 CCS values across ion species.

Figure S3. The number of compounds detected in positive and negative ion modes.

Figure S4. Distribution of 7017 CCS values across ion species after consolidation.

Table S1. Distribution of 4170 compounds across 17 super classes

Table S2. Comparison between the CCS values measured via DTIMS and TWIMS devices.

Table S3. Distributions of CCS records across different ionic species.

Table S4. Distribution of RSDs (%) of repeated CCS measurements across different ion species

Table S5. Several published CCS prediction tools.

Table S6. Several isomeric pairs and their CCS values found in literature.

Table S7. Comparison of the CCS measurements of 12 molecules obtained from FDA approved drugs profliing CCS library and Celma et al.

Introduction of DTIMS, TWIMS and TIMS.

Three Reasons Leading to High CCS Deviations.

Commonly Used Molecular Descriptors for CCS Prediction.

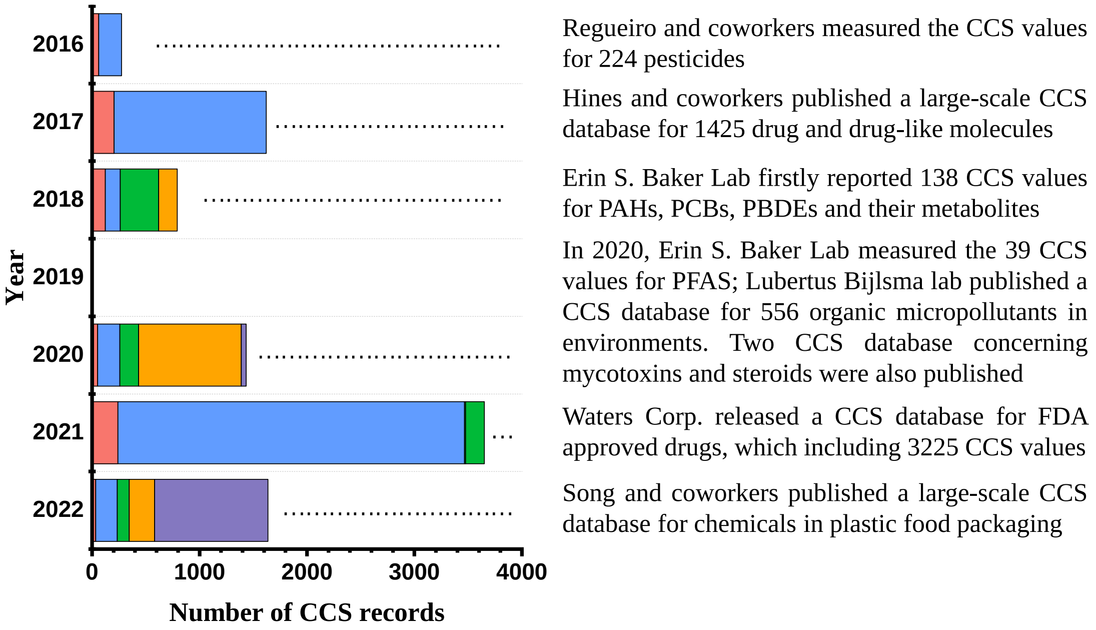

Figure S1. Distributions of the yearly publication of CCS records and the contributions of the main research groups. Different color in the same year indicate the contributions of different groups.

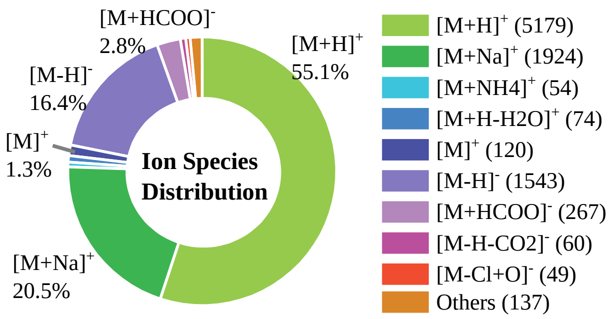

Figure S2. Distribution of 9407 CCS values across ion species.

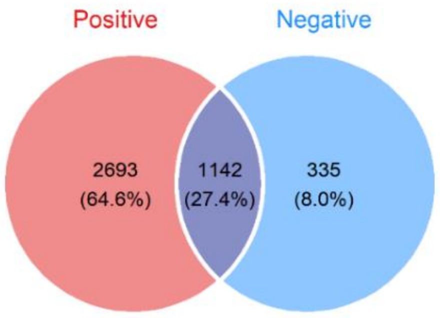

Figure S3. The number of compounds detected in positive and negative ion modes.

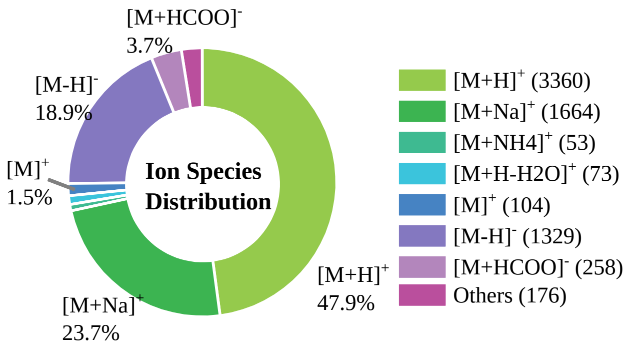

Figure S4. Distribution of 7017 CCS values across ion species after consolidation.

Table S1. Distribution of 4170 compounds across 17 super classes

Superclass	Count	Proportion (%)
Benzenoids	1247	29.90
Organoheterocyclic compounds	1084	26.00
Lipids and lipid-like molecules	541	12.97
Organic acids and derivatives	411	9.86
Phenylpropanoids and polyketides	317	7.60
Organic oxygen compounds	244	5.85
Alkaloids and derivatives	102	2.45
Organic nitrogen compounds	85	2.04
Organohalogen compounds	51	1.22
Nucleosides, nucleotides, and analogues	42	1.01
Organosulfur compounds	16	0.38
Organophosphorus compounds	11	0.26
Lignans, neolignans and related compounds	10	0.24
Organometallic compounds	4	0.10
Hydrocarbon derivatives	3	0.07
Hydrocarbons	1	0.02
Organic 1,3-dipolar compounds	1	0.02
**Total**	**4170**	**100**

Table S2. Comparison between the CCS values measured via DTIMS and TWIMS devices.

Adducts	Counts	Max error(%)	Min error(%)	Median error (%)	Mean error (%)	< 2%	< 5%
$[M+H]^+$	480	6.2	-6.1	-0.3	-0.4	417 (86.9%)	476 (99.2%)
$[M+Na]^+$	38	5.2	-4.2	-0.5	-0.4	28 (73.7%)	37 (97.4%)
$[M]^+$	14	1.2	-2.4	-1,4	-1.2	12 (85.7)	14 (100%)
$[M-H]^-$	48	0.7	-3.5	-1.9	-1.7	25 (52.1%)	48 (100%)
Total	580	6.2	-6.1	-0.5	-0.5	482 (83.1%)	5 (99.1%)

Table S3. Distributions of CCS records across different ionic species.

		**9407** CCS collected		**7017** CCS after normalization	
	Adducts	Number of CCS	Proportion (%)	Number of CCS	Proportion (%)
positive	$[M+H]^+$	5179	55.1	3360	47.9
	$[M+Na]^+$	1924	20.5	1664	23.7
	$[M+NH_4]^+$	54	0.6	53	0.8
	$[M+H-H_2O]^+$	74	0.8	73	1.0
	$[M]^{+\bullet}$	120	1.3	104	1.5
	$[M+K]^+$	33	0.4	32	0.5
	$[M+H-NH_3]^+$	9	0.1	9	0.1
	$[M-Na+2H]^+$	5	0.1	5	0.1
	$[M-Cl]^+$	11	0.1	11	0.2
	**Total**	**7409**	**78.8**	**5311**	**75.7**
negative	$[M-H]^-$	1543	16.4	1329	18.9
	$[M+HCOO]^-$	267	2.8	258	3.7
	$[M-H-CO_2]^-$	60	0.6	31	0.4
	$[M-Cl+O]^-$	49	0.5	26	0.4
	$[M+CH_3COO]^-$	23	0.2	22	0.3
	$[M+Cl]^-$	24	0.3	24	0.3
	$[M-Br+O]^-$	32	0.3	16	0.2
	**Total**	**1998**	**21.2**	**1706**	**24.3**

Table S4. Distribution of RSDs (%) of repeated CCS measurements across different ion species

Ion type	Adducts	Count	Max	Min	Median	Mean	< 2%	< 5%
Positive	$[M+H]^+$	1059	10.98	0	0.58	0.87	92.3%	98.0%
	$[M+Na]^+$	214	7.90	0	0.64	0.82	96.7%	98.1%
	$[M]^+$	16	1.74	0.02	0.93	0.91	100%	100%
	$[M+H-H2O]^+$	1	2.71	2.71	2.71	2.71	0	100%
	Total	**1290**	**10.98**	**0**	**0.59**	**0.86**	**93.0%**	**98.1%**
Negative	$[M-H]^-$	177	7.64	0.01	0.76	0.84	96.6%	99.4%
	$[M+HCOO]^-$	9	1.25	0	0.85	0.69	100%	100%
	$[M-Cl+O]^-$	23	0.26	0.22	0.24	0.24	100%	100%
	$[M-Br+O]^-$	16	0.26	0.22	0.25	0.24	100%	100%
	$[M-H-CO2]^-$	16	3.03	0	0.23	0.37	93.4%	100%
	Total	**241**	**7.64**	**0**	**0.30**	**0.70**	**97.1%**	**99.6%**
Total ions		**1531**	**10.98**	**0**	**0.57**	**0.84**	**93.7%**	**98.3%**

Table S5. Several published CCS prediction tools.

Tools	Training data	Algorithm	Adducts	Reference
CCSondemand	7325 $^{TW}CCS_{N2}$ values from 3775 compounds	XGBoost	$[M+H]^+$, $[M+Na]^+$, $[M+K]^+$, $[M-H]^-$, $[M+HCOO]^-$,	Broeckling et al.[1]
AllCCS	5119 CCS values from 2193 compounds	SVM	$[M+H]^+$, $[M+Na]^+$, $[M+NH_4]^+$, $[M+H-H_2O]^+$, $[M-H]^-$, $[M+HCOO]^-$, $[M+Na-2H]^-$	Zhou et al.[2]
CCSbase	7405 CCS entries from 3526 small molecules, 1041 lipids, 91 peptides, 84 carbohydrates	SVM	$[M+H]^+$, $[M+Na]^+$, $[M+NH_4]^+$, $[M+K]^+$, $[M-H]^-$, $[M+Na-2H]^-$, $[M]^{+\bullet}$, $[M]^-$	Ross et al.[3]
DeepCCS	2439 CCS values	DNN	$[M+H]^+$, $[M+Na]^+$, $[M-H]^-$, $[M-2H]^{2-}$	Plante et al.[4]
CCSP 2.0	1546 CCS values	SVM	$[M+H]^+$, $[M+Na]^+$, $[M-H]^-$	Rainey et al.[5]
LipidCCS	458 CCS values	SVM	$[M+H]^+$, $[M+Na]^+$, $[M+NH_4]^+$, $[M-H]^-$, $[M+HCOO]^-$	Zhou et al.[6]
MetCCS	796 CCS values	SVM	$[M+H]^+$, $[M+Na]^+$, $[M+H-H_2O]^+$, $[M-H]^-$, $[M+Na-2H]^-$	Zhou et al.[7]
CCS prediction for pesticides	205 CCS values of pesticides	ANN	$[M+H]^+$, $[M+Na]^+$	Bijlsma et al.[8]
CCS prediction for drugs	357 CCS values of pharmaceuticals, drugs	ANN	$[M+H]^+$	Mollerup et al.[9]
CCS prediction for plastic chemicals	1721 CCS values of chemicals in plastic products	SVM	$[M+H]^+$, $[M+Na]^+$	Song et al.[10]
Emerging contaminants	895 CCS values of emerging contaminants	MARS	$[M+H]^+$, $[M+Na]^+$, $[M-H]^-$	Celma et al.[11]
PFAS, PAHs, PCBs, PBDEs	202 CCS values of PFAS, PAHs, PCBs, and PBDEs	Linear SVM	$[M+H]^+$, $[M-H]^-$, $[M-H-CO_2]^-$, $[M-Br+O]^-$, $[M-Cl+O]^-$,	Foster et al.[12]
CCS prediction for	56 CCS values of phenolics	PLS	$[M-H]^-$	Gonzales et al.[13]

Tools	Training data	Algorithm	Adducts	Reference
phenolics				
CCS prediction for metabolites	1226 CCS values of metabolites	SVM	$[M+H]^+$, $[M-H]^-$	Asef et al.[14]

Note: XGBoost: extreme gradient boosting; SVM: support vector machine; DNN: deep neural network; ANN: artificial neural networks; PLS: partial least squares regression; MARS: multiple adaptive regression splines; PFAS: per- and polyfluoroalkyl substances; PAHs: polycyclic aromatic hydrocarbons; PCBs: polychlorinated biphenyls; PBDEs: polybrominated diphenyl ethers.

Table S6. Several isomeric pairs and their CCS values found in literature.

Isomer pairs	Adducts	Experimental CCS (Å^2)	Reference
morphine and piperine	$[M+H]^+$	164.0/176.2	Lian et al.[15]
15-acetyldeoxynivalenol and 3-acetyldeoxynivalenol<br>T2 alpha-glucoside and T2 beta-glucoside	$[M+Na]^+$<br>$[M+CH3COO]^-$	176.7/183.4<br>252.9/264.8	Righetti et al.[16]
Alternariol 9-glucuronide, Alternariol 3-glucuronide and Alternariol 7-glucuronide;	$[M-H]^-$	197.1/203.8/213.8	Righetti et al.[17]
ethiofencarb sulfoxide<br>methiocarb sulfoxide;<br>testosterone glucuronide<br>epitestosterone glucuronide;<br>ketamine and 4'-Chloro-alpha-pyrrolidinopropiophenone	$[M+H]^+$	146.5/156.9<br>221.5/204.7<br>148.9/154.0	Celma et al.[18]
ipconazole and tebufenpyrad	$[M+H]^+$	163.6/193.4	Regueiro et al.[19]
Testosterone, DHEA and Epitestosterone;<br>Androsterone and Epiandrosterone;<br>11-Deoxycortisol and Corticosterone	$[2M+Li]^+$	283.8/272.7/275.7<br>253.7/273.5<br>295.1/305.6	Rister et al.[20]
Ergosine and ergosinine<br>Ergotamine and ergotaminine<br>Ergokryptine and ergokryptinine<br>Ergocristine and ergocristinine	$[M+Na]^+$	234.2/226.5<br>236.2/227.2<br>239.1/233.7<br>241.7/233.9	Carbonell-Rozas et al.[21]
Diclofenac β-1-O-acyl glucuronide and 2-, 3-, and 4-O-acyl isomers.	$[M+H]^+$	205.3/201.1/200.9/199.7	Higton et al.[22]
Linear PFOS and 1m-PFOS;<br>Linear PFHxS and 1m-PFHxS	$[M-H]^-$	162.3/157.9<br>145.8/141.6	Mu et al.[23]

Isomer pairs	Adducts	Experimental CCS (Å^2)	Reference
tri-m-tolyl phosphate<br>tri-o-tolyl phosphate<br>tri-p-tolyl phosphate	$[M+H]^+$<br>$[M+Na]^+$	188.6/182.4/190.0<br>198.6/192.4/200.0	Belva et al.[24]
PA 6 dimer and PA66 monomer;<br>PA6 tetramer and PA66 dimer;<br>PA6 hexamer and PA66 trimer	$[M+H]^+$	150.4/157.8<br>209.6/214.7<br>255.1/261.1	Schweighuber et al.[25]
Pregnenolone and 5α-dihydroprogesterone;	$[M+H]^+$	176.7/191.4	Chouinard et al.[26]
Aldosterone and cortisone	$[M+Na]^+$	197.7/211.7	

Table S7. Comparison of the CCS measurements of 12 molecules obtained from FDA approved drugs profiling CCS library[27] and Celma et al.[28]

Compounds	PubChem CID	CCS from FDA	CCS from Celma et al.	Variations
Flumethasone	16490	191.57	195.21	3.64
Clindamycin	446598	197.69	204.41	6.72
Lincomycin	3000540	196.77	202.26	5.49
Amoxicillin	33613	183.83	188.68	4.85
Mefenamic acid	4044	157.25	161.30	4.05
Metaproterenol	4086	151.49	155.30	3.81
Butylparaben	7184	146.52	150.14	3.62
Fenofibric acid	64929	181.44	185.72	4.28
Salbutamol	2083	162.83	166.60	3.77
Flubendazole	35802	173.42	177.36	3.94
Cefotaxime	5742673	200.26	204.79	4.53
Telmisartan	65999	237.16	242.47	5.31

Introduction of DTIMS, TWIMS and TIMS

DTIMS is a classic IMS technique, the design of stacked ring electrodes to maintain a uniform field can be traced back to 1930s,[29] and the first commercialized DTIMS technique was introduced in 1970s.[30] The drift tube of DTIMS is made from a series of stacked-ring electrodes that can provide a uniform, static and weak electric field, normally in the range of 13–18 V/cm.[31] The ionized molecules are pushed by an

electric field force into the drift tube and collide with stationary buffer gas (normally nitrogen or helium, but also carbon dioxide). Molecules with smaller cross-section traverse the drift tube more quickly than the molecules with larger cross-section since they have fewer collisions with buffer gas. The time taken to traverse the drift cell is called drift time (t_d), which can be used to calculate the mobility constant *K*, and further the CCS values of ions, using the Mason-Schamp equation.[32] The ability of directly calculating CCS values from first principles seems the most attractive advantage of DTIMS. The CCS values obtained using this method are considered more accurate and are always used for the CCS calibration of other types of IMS.[33] The resolving power (*R*p) of DTIMS changes based on temperature, length and pressure, the DTIMS 6560 commercialized by Agilent Technologies can provide a *R*p ~60.[34] Increasing the drift tube length can theoretically enhance *R*p. A cyclic DTIMS devices, produced by Clemmer group, can achieve *R*p ~1000 when the ions travel 100 cycles with an effective distance of about 180 m.[35] In addition, DTIMS coupled with high resolution demultiplexing (HRdm) technique, also achieve *R*p between 180 to 250.[36] One disadvantage of DTIMS involves the low duty cycle, which leads to reduced sensitivity of the detection. More detailed discussions about the advantages and disadvantages of DTIMS can be seen in Dodds et al.[37]

TWIMS is another widely used IMS technique, which was first commercialized by Waters Corporation in 2006.[38] TWIMS consists of a stacked ring ion guide where a series of voltage pulses are applied, creating a travelling wave that ions can 'surf' along.[39] A radio frequency voltage is applied to adjacent ring electrodes to radially

confine ions, in order to maintain high transmission. Unlike DTIMS in which CCS values can be directly calculated from drift time, in TWIMS, the IMS cell has to be calibrated with a set of reference compounds with known CCS values. Currently, Rp around 40-50 CCS/ΔCCS have been reported for some commercial TWIMS platforms,[40] however, an extremely high Rp of 750 was achieved for ions traveling 100 passes in a cyclic TWIMS platform.[41].

TIMS was commercialized by Bruker Daltonics in 2011.[42] Unlike conventional DTIMS and TWIMS where the ions move in a stationary buffer gas forced by a low electric field, TIMS holds the ions stationary in a moving buffer gas by applying an electric field in the opposite direction. The field strength in TIMS is slowly decreased allowing the ejection of the ions with specific mobility.[43] It has been reported that TIMS can offer a R_p exceeding 250.[44] In TIMS, experimental CCS values are obtained by applying an appropriate calibration derived using reference compounds with known CCS values.

Three Reasons Leading to High CCS Deviations

Presence of Protomers. The presence of protomers can lead to high CCS errors since different protonation sites result in the molecules having a different shape and size. This can be demonstrated by the multiple CCS values that have been determined for the $[M + H]^+$ adduct of enoxacin. The study of Hines and coworkers reports two different CCS values for enoxacin of 168.1 and 184.7 Å^2,[45] two other CCS values for this compound were also found in the published data: 185.4 Å^2 from the FDA approved

drugs profiling CCS library,[27] and 178.0 Å^2 from Tejada-Casado et al.[46] Together, the four CCS values resulted in an RSD value of 5.12%. Only single CCS values were obtained for enoxacin in the latter two publications because the ion mobility data was not sufficiently resolved, given the relatively low resolving power of current commercial IMS techniques. Some high RSDs values of other CCS values can also be explained by the presence of protomers, for example, Hines et al.[45] also report two CCS values for the $[M + H]^+$ adduct of bacampicillinm (199.0 and 220.8 Å^2), two CCS values for the $[M + H]^+$ adduct of temefos (187.6 and 203.2 Å^2).

IMS separation of protomers for pesticides and pharmaceuticals has also been reported in other literature.[47-50] One advantage of protomer formation is that multiple CCS values can be obtained for each molecule (generally with CCS deviations higher than 2%), these values can be utilized to further add the specificity for chemical identifications in screening analyses. In addition, the fragmentation of protomers of the same compound can be different and the in-depth study of their mass spectra can help us to gain more understanding of ionization and dissociation mechanisms occurring in the mass spectrometer.[49] It is important to note though that the presence of protomers can complicate the data treatment of TA and SSA. True identifications may be missed if a compound exhibits multiple CCS values and only one of the values is targeted.

Calibration Approach. The fact that the calibration standards for different TWIMS platforms can vary is another reason that can lead to high CCS discrepancies.[33] As an example of this, three CCS values were found for the $[M - H]^-$ adduct of captopril, two of which were 146.6 Å^2 [27] and 148.1 Å^2,[51] while the third, in the study by Hines et

al.,[45] had a value of 141.4 Å^2, which is much lower than the other two values. In addition, two CCS values were found for the $[M + H]^+$ adduct of ferulic acid: 128.8[45] and 141.1 Å^2,[51] with the high CCS deviation occurring, once again, between Hines et al.[45] and Song et al.[51] In their study, Song et al.[51] used the Major Mix IMS/ToF calibration kit (p/n 186008113) from Waters (Manchester, UK) for the CCS calibration. This mixture is also always used for the CCS calibration of other TWIMS platforms from Waters Corporation, and contains polyalanine, Ultramark, and drug-like compounds. Two additional fluoroalkanoic acids are added into the calibration mixture to calibrate negative ion mode. In the work of Hines and coworkers,[45] the TWIMS device was calibrated using polyalanine and nine drug-like compounds, but the nine drug-like compounds were not the same as those used in the work of Song et al.[51] The high CCS variation due to calibration issues can be decreased or eliminated by adopting a consistent CCS calibration approach for TWIMS instrumentation.

In some cases, high CCS variations still occur even if the same CCS calibration procedures are applied to the TWIMS platforms. Table S7 shows the CCS values of 12 compounds obtained from two publications. Generally, the CCS values in the FDA approved drugs profiling CCS library[27] were ~5 Å^2 lower than those obtained for the same compounds in the study of Celma et al.[28] and both studies used the same CCS calibration approach. Therefore, these CCS variations indicates that an improved CCS calibration approach, producing higher reproducibility for CCS measurements, needs to be proposed for the current TWIMS platforms.

Post-IMS Dissociation of Noncovalent Clusters. Another reason for high CCS

variations is the post-IMS dissociation of noncovalent clusters. In some cases, a noncovalent cluster can be formed in the ion source and then travel in IMS cell, thus producing an elevated CCS value due to its larger size compared with target ion. This phenomenon was well depicted and explained in the study of Song and coworkers,[51] in which five CCS values were obtained for the $[M - H]^-$ adduct of triclosan. The actual CCS value of triclosan was 157.4 Å^2, this value matched the CCS values from other publications.[24, 27, 28] The other four elevated CCS values obtained for triclosan were 177.5, 203.9, 227.5, and 257.0 Å^2, all of which are much higher than the actual value. In this CCS compendium, there is a high variation of the CCS values of protonated chlorpyrifos (RSD value of 10.98%) and may arise from the post-IMS dissociation of noncovalent clusters. Four CCS values were found for chlorpyrifos from publications,[19, 28, 45, 52] three of which ranged from 163.0 to 165.0 Å^2,[19, 28, 52] while the fourth, in the study of Hines et al.,[45] had a value of 201.7 Å^2, depicting a variation of ~38 Å^2 from other three values. In many cases the sources of CCS variations can be difficult to pinpoint. For example, five experimental CCS values were found for the $[M + H]^+$ adduct of dexamethasone, four of which ranged from 183.5 to 191.6 Å^2,[27, 45, 51, 53] while the fifth, from the study by Plachka et al.[54], had a value of 216.4 Å^2. Additionally, the study of Plachka et al.[54] also produced a relatively higher CCS value (216.2 Å^2) for the $[M + H]^+$ of betamethasone, compared to the values (186.7-194.2 Å^2) obtained from other literature.[27, 28, 45, 53] As these two compounds contain multiple protonation sites in their structures, these high CCS discrepancies could be caused by the formation of protomers or the post-IMS dissociation of noncovalent clusters.

The CCS records with high discrepancies are likely to be removed by using CCS versus mass trend lines for each super class with a predefined predictive interval,[2] however, this method has a risk of removing correct CCS records that are far from the trend lines. The use of Grubbs' test is also an alternative approach to remove CCS outliers, however, this method is not applicable in cases where two significantly different CCS values are published for the same adducts of compounds. Another promising approach of removing potential outliers is by using CCS prediction tools, some suspect outliers can be easily detected by comparing their predicted and measured CCS values, this approach was adopted in the study of Plante et al.,[4] which enabled the successful identification of five outliers.

Commonly Used Molecular Descriptors for CCS Prediction

Currently, there is no universal agreement on which molecular descriptor is essential for predicting the CCS values, however, certain descriptors have been frequently utilized in various CCS prediction models. Atom molar refractivity (AMR) is a constitutional descriptor, it represents the volume of the molecules for a radiation of infinite wavelength,[55] and is used to predict the CCS in MetCCS,[7] AllCCS,[2] Song et al.[10] and Celma et al.[11] The descriptors calculating the polarizability of molecules are also often used for CCS prediction.[5, 7, 10] In CCSP 2.0,[5] the most prominent descriptor classes used in SVR models are autocorrelation of topological structure (ATS) descriptors and Barysz Matrix descriptors, which are also related to the molecular polarizability. Some constitutional descriptor, such as the number of atoms (nAtom),

the number of atoms in the longest aliphatic chain (nAtomLAC), the number of atoms in the largest chain (nAtomLC), molecular weight, are also contribute to CCS prediction.[2, 10] The topological descriptors, such as Kier & Hall Chi path indices, kappa shape index, Wiener index, are constantly utilized in AllCCS,[2] CCSP 2.0,[5] Gonzales et al.[13] and Mollerup et al.[9]

References:

1. Broeckling, C. D.; Yao, L.; Isaac, G.; Gioioso, M.; Ianchis, V.; Vissers, J. P. C., Application of Predicted Collisional Cross Section to Metabolome Databases to Probabilistically Describe the Current and Future Ion Mobility Mass Spectrometry. *J. Am. Soc. Mass Spectrom.* **2021,** *32*, (3), 661-669.
2. Zhou, Z.; Luo, M.; Chen, X.; Yin, Y.; Xiong, X.; Wang, R.; Zhu, Z. J., Ion mobility collision cross-section atlas for known and unknown metabolite annotation in untargeted metabolomics. *Nat. Commun.* **2020,** *11*, (1), 4334.
3. Ross, D. H.; Cho, J. H.; Xu, L., Breaking Down Structural Diversity for Comprehensive Prediction of Ion-Neutral Collision Cross Sections. *Anal. Chem.* **2020,** *92*, (6), 4548-4557.
4. Plante, P. L.; Francovic-Fontaine, E.; May, J. C.; McLean, J. A.; Baker, E. S.; Laviolette, F.; Marchand, M.; Corbeil, J., Predicting Ion Mobility Collision Cross-Sections Using a Deep Neural Network: DeepCCS. *Anal. Chem.* **2019,** *91*, (8), 5191-5199.
5. Rainey, M. A.; Watson, C. A.; Asef, C. K.; Foster, M. R.; Baker, E. S.; Fernandez, F. M., CCS Predictor 2.0: An Open-Source Jupyter Notebook Tool for Filtering Out False Positives in Metabolomics. *Anal. Chem.* **2022,** *94*, (50), 17456-17466.
6. Zhou, Z.; Tu, J.; Xiong, X.; Shen, X.; Zhu, Z. J., LipidCCS: Prediction of Collision Cross-Section Values for Lipids with High Precision To Support Ion Mobility-Mass Spectrometry-Based Lipidomics. *Anal. Chem.* **2017,** *89*, (17), 9559-9566.
7. Zhou, Z.; Shen, X.; Tu, J.; Zhu, Z. J., Large-Scale Prediction of Collision Cross-Section Values for Metabolites in Ion Mobility-Mass Spectrometry. *Anal. Chem.* **2016,** *88*, (22), 11084-11091.
8. Bijlsma, L.; Bade, R.; Celma, A.; Mullin, L.; Cleland, G.; Stead, S.; Hernandez, F.; Sancho, J. V., Prediction of Collision Cross-Section Values for Small Molecules: Application to Pesticide Residue Analysis. *Anal. Chem.* **2017,** *89*, (12), 6583-6589.
9. Mollerup, C. B.; Mardal, M.; Dalsgaard, P. W.; Linnet, K.; Barron, L. P., Prediction of collision cross section and retention time for broad scope screening in gradient reversed-phase liquid

chromatography-ion mobility-high resolution accurate mass spectrometry. *J. Chromatogr. A* **2018,** *1542*, 82-88.
10. Song, X. C.; Dreolin, N.; Canellas, E.; Goshawk, J.; Nerin, C., Prediction of Collision Cross-Section Values for Extractables and Leachables from Plastic Products. *Environ. Sci. Technol.* **2022,** *56*, (13), 9463-9473.
11. Celma, A.; Bade, R.; Sancho, J. V.; Hernandez, F.; Humphries, M.; Bijlsma, L., Prediction of Retention Time and Collision Cross Section (CCS(H+), CCS(H-), and CCS(Na+)) of Emerging Contaminants Using Multiple Adaptive Regression Splines. *J. Chem. Inf. Model.* **2022,** *62*, (22), 5425-5434.
12. Foster, M.; Rainey, M.; Watson, C.; Dodds, J. N.; Kirkwood, K. I.; Fernandez, F. M.; Baker, E. S., Uncovering PFAS and Other Xenobiotics in the Dark Metabolome Using Ion Mobility Spectrometry, Mass Defect Analysis, and Machine Learning. *Environ. Sci. Technol.* **2022,** *56*, (12), 9133-9143.
13. Gonzales, G. B.; Smagghe, G.; Coelus, S.; Adriaenssens, D.; De Winter, K.; Desmet, T.; Raes, K.; Van Camp, J., Collision cross section prediction of deprotonated phenolics in a travelling-wave ion mobility spectrometer using molecular descriptors and chemometrics. *Anal. Chim. Acta* **2016,** *924*, 68-76.
14. Asef, C. K.; Rainey, M. A.; Garcia, B. M.; Gouveia, G. J.; Shaver, A. O.; Leach, F. E., 3rd; Morse, A. M.; Edison, A. S.; McIntyre, L. M.; Fernandez, F. M., Unknown Metabolite Identification Using Machine Learning Collision Cross-Section Prediction and Tandem Mass Spectrometry. *Anal. Chem.* **2023,** *95*, (2), 1047-1056.
15. Lian, R.; Zhang, F.; Zhang, Y.; Wu, Z.; Ye, H.; Ni, C.; Lv, X.; Guo, Y., Ion mobility derived collision cross section as an additional measure to support the rapid analysis of abused drugs and toxic compounds using electrospray ion mobility time-of-flight mass spectrometry. *Anal. Methods* **2018,** *10*, (7), 749-756.
16. Righetti, L.; Dreolin, N.; Celma, A.; McCullagh, M.; Barknowitz, G.; Sancho, J. V.; Dall'Asta, C., Travelling Wave Ion Mobility-Derived Collision Cross Section for Mycotoxins: Investigating Interlaboratory and Interplatform Reproducibility. *J. Agric. Food Chem.* **2020,** *68*, (39), 10937-10943.
17. Righetti, L.; Fenclova, M.; Dellafiora, L.; Hajslova, J.; Stranska-Zachariasova, M.; Dall'Asta, C., High resolution-ion mobility mass spectrometry as an additional powerful tool for structural characterization of mycotoxin metabolites. *Food Chem.* **2018,** *245*, 768-774.
18. Celma, A.; Ahrens, L.; Gago-Ferrero, P.; Hernández, F.; López, F.; Lundqvist, J.; Pitarch, E.; Sancho, J. V.; Wiberg, K.; Bijlsma, L., The relevant role of ion mobility separation in LC-HRMS based screening strategies for contaminants of emerging concern in the aquatic environment. *Chemosphere* **2021,** *280*, 130799.
19. Regueiro, J.; Negreira, N.; Berntssen, M. H., Ion-Mobility-Derived Collision Cross Section as an Additional Identification Point for Multiresidue Screening of Pesticides in Fish Feed. *Anal. Chem.* **2016,** *88*, (22), 11169-11177.
20. Rister, A. L.; Dodds, E. D., Liquid chromatography-ion mobility spectrometry-mass spectrometry analysis of multiple classes of steroid hormone isomers in a mixture. *J. Chromatogr. B* **2020,** *1137*, 121941.
21. Carbonell-Rozas, L.; Hernández-Mesa, M.; Righetti, L.; Monteau, F.; Lara, F. J.; Gámiz-Gracia, L.; Bizec, B. L.; Dall'Asta, C.; García-Campaña, A. M.; Dervilly, G., Ion mobility-mass spectrometry to extend analytical performance in the determination of ergot alkaloids in cereal samples. *J. Chromatogr. A* **2022,** *1682*, 463502.
22. Higton, D.; Palmer, M. E.; Vissers, J. P. C.; Mullin, L. G.; Plumb, R. S.; Wilson, I. D., Use of Cyclic

Ion Mobility Spectrometry (cIM)-Mass Spectrometry to Study the Intramolecular Transacylation of Diclofenac Acyl Glucuronide. *Anal. Chem.* **2021,** *93*, (20), 7413-7421.
23. Mu, H.; Wang, J.; Chen, L.; Hu, H.; Wang, J.; Gu, C.; Ren, H.; Wu, B., Identification and characterization of diverse isomers of per- and polyfluoroalkyl substances in Chinese municipal wastewater. *Water Res.* **2023,** *230*, 119580.
24. Belova, L.; Caballero-Casero, N.; van Nuijs, A. L. N.; Covaci, A., Ion Mobility-High-Resolution Mass Spectrometry (IM-HRMS) for the Analysis of Contaminants of Emerging Concern (CECs): Database Compilation and Application to Urine Samples. *Anal. Chem.* **2021,** *93*, (16), 6428-6436.
25. Schweighuber, A.; Fischer, J.; Buchberger, W., Differentiation of Polyamide 6, 6.6, and 12 Contaminations in Polyolefin-Recyclates Using HPLC Coupled to Drift-Tube Ion-Mobility Quadrupole Time-of-Flight Mass Spectrometry. *Polymers* **2021,** *13*, (12), 2032.
26. Chouinard, C. D.; Beekman, C. R.; Kemperman, R. H. J.; King, H. M.; Yost, R. A., Ion mobility-mass spectrometry separation of steroid structural isomers and epimers. *Int. J. Ion Mobil. Spectrom.* **2016,** *20*, (1-2), 31-39.
27. Waters Corporation., FDA approved drugs profiling CCS library. In https://marketplace.waters.com/apps/338581/fda-approved-drugs#!overview (accessed 29 July, 2023).
28. Celma, A.; Sancho, J. V.; Schymanski, E. L.; Fabregat-Safont, D.; Ibanez, M.; Goshawk, J.; Barknowitz, G.; Hernandez, F.; Bijlsma, L., Improving Target and Suspect Screening High-Resolution Mass Spectrometry Workflows in Environmental Analysis by Ion Mobility Separation. *Environ. Sci. Technol.* **2020,** *54*, (23), 15120-15131.
29. Tyndall, A. M.; Powell, C. F.; Chattock, A. P., The mobility of positive ions in helium. Part I.—Helium ions. *Proceedings of the Royal Society of London. Series A, Containing Papers of a Mathematical and Physical Character* **1931,** *134*, (823), 125-136.
30. Cohen, M. J.; Karasek, F. W., Plasma Chromatography™—A New Dimension for Gas Chromatography and Mass Spectrometry. *J. Chromatogr. Sci.* **1970,** *8*, (6), 330-337.
31. D'Atri, V.; Causon, T.; Hernandez-Alba, O.; Mutabazi, A.; Veuthey, J. L.; Cianferani, S.; Guillarme, D., Adding a new separation dimension to MS and LC-MS: What is the utility of ion mobility spectrometry? *J. Sep. Sci.* **2018,** *41*, (1), 20-67.
32. Mason, E. A.; Schamp, H. W., Mobility of gaseous lons in weak electric fields. *Ann. Phys.* **1958,** *4*, (3), 233-270.
33. Hines, K. M.; May, J. C.; McLean, J. A.; Xu, L., Evaluation of Collision Cross Section Calibrants for Structural Analysis of Lipids by Traveling Wave Ion Mobility-Mass Spectrometry. *Anal. Chem.* **2016,** *88*, (14), 7329-7336.
34. Dodds, J. N.; May, J. C.; McLean, J. A., Investigation of the Complete Suite of the Leucine and Isoleucine Isomers: Toward Prediction of Ion Mobility Separation Capabilities. *Anal. Chem.* **2017,** *89*, (1), 952-959.
35. Glaskin, R. S.; Ewing, M. A.; Clemmer, D. E., Ion Trapping for Ion Mobility Spectrometry Measurements in a Cyclical Drift Tube. *Anal. Chem.* **2013,** *85*, (15), 7003-7008.
36. May, J. C.; Knochenmuss, R.; Fjeldsted, J. C.; McLean, J. A., Resolution of Isomeric Mixtures in Ion Mobility Using a Combined Demultiplexing and Peak Deconvolution Technique. *Anal. Chem.* **2020,** *92*, (14), 9482-9492.
37. Dodds, J. N.; Baker, E. S., Ion Mobility Spectrometry: Fundamental Concepts, Instrumentation, Applications, and the Road Ahead. *J. Am. Soc. Mass Spectrom.* **2019,** *30*, (11), 2185-2195.
38. Pringle, S. D.; Giles, K.; Wildgoose, J. L.; Williams, J. P.; Slade, S. E.; Thalassinos, K.; Bateman,

R. H.; Bowers, M. T.; Scrivens, J. H., An investigation of the mobility separation of some peptide and protein ions using a new hybrid quadrupole/travelling wave IMS/oa-ToF instrument. *Int. J. Mass Spectrom.* **2007,** *261*, (1), 1-12.
39. Shvartsburg, A. A.; Smith, R. D., Fundamentals of Traveling Wave Ion Mobility Spectrometry. *Anal. Chem.* **2008,** *80*, (24), 9689-9699.
40. Dodds, J. N.; May, J. C.; McLean, J. A., Correlating Resolving Power, Resolution, and Collision Cross Section: Unifying Cross-Platform Assessment of Separation Efficiency in Ion Mobility Spectrometry. *Anal. Chem.* **2017,** *89*, (22), 12176-12184.
41. Giles, K.; Ujma, J.; Wildgoose, J.; Pringle, S.; Richardson, K.; Langridge, D.; Green, M., A Cyclic Ion Mobility-Mass Spectrometry System. *Anal. Chem.* **2019,** *91*, (13), 8564-8573.
42. Fernandez-Lima, F.; Kaplan, D. A.; Suetering, J.; Park, M. A., Gas-phase separation using a trapped ion mobility spectrometer. *Int. J. Ion Mobil. Spectrom.* **2011,** *14*, (2-3), 93-98.
43. Ridgeway, M. E.; Lubeck, M.; Jordens, J.; Mann, M.; Park, M. A., Trapped ion mobility spectrometry: A short review. *Int. J. Mass Spectrom.* **2018,** *425*, 22-35.
44. Silveira, J. A.; Ridgeway, M. E.; Park, M. A., High resolution trapped ion mobility spectrometery of peptides. *Anal. Chem.* **2014,** *86*, (12), 5624-5627.
45. Hines, K. M.; Ross, D. H.; Davidson, K. L.; Bush, M. F.; Xu, L., Large-Scale Structural Characterization of Drug and Drug-Like Compounds by High-Throughput Ion Mobility-Mass Spectrometry. *Anal. Chem.* **2017,** *89*, (17), 9023-9030.
46. Tejada-Casado, C.; Hernandez-Mesa, M.; Monteau, F.; Lara, F. J.; Olmo-Iruela, M. D.; Garcia-Campana, A. M.; Le Bizec, B.; Dervilly-Pinel, G., Collision cross section (CCS) as a complementary parameter to characterize human and veterinary drugs. *Anal. Chim. Acta* **2018,** *1043*, 52-63.
47. Boschmans, J.; Jacobs, S.; Williams, J. P.; Palmer, M.; Richardson, K.; Giles, K.; Lapthorn, C.; Herrebout, W. A.; Lemiere, F.; Sobott, F., Combining density functional theory (DFT) and collision cross-section (CCS) calculations to analyze the gas-phase behaviour of small molecules and their protonation site isomers. *Analyst* **2016,** *141*, (13), 4044-4054.
48. Warnke, S.; Seo, J.; Boschmans, J.; Sobott, F.; Scrivens, J. H.; Bleiholder, C.; Bowers, M. T.; Gewinner, S.; Schollkopf, W.; Pagel, K.; von Helden, G., Protomers of benzocaine: solvent and permittivity dependence. *J. Am. Chem. Soc.* **2015,** *137*, (12), 4236-4242.
49. McCullagh, M.; Giles, K.; Richardson, K.; Stead, S.; Palmer, M., Investigations into the performance of travelling wave enabled conventional and cyclic ion mobility systems to characterise protomers of fluoroquinolone antibiotic residues. *Rapid Commun. Mass Spectrom.* **2019,** *33 Suppl 2*, 11-21.
50. Hinnenkamp, V.; Klein, J.; Meckelmann, S. W.; Balsaa, P.; Schmidt, T. C.; Schmitz, O. J., Comparison of CCS Values Determined by Traveling Wave Ion Mobility Mass Spectrometry and Drift Tube Ion Mobility Mass Spectrometry. *Anal. Chem.* **2018,** *90*, (20), 12042-12050.
51. Song, X. C.; Canellas, E.; Dreolin, N.; Goshawk, J.; Nerin, C., A Collision Cross Section Database for Extractables and Leachables from Food Contact Materials. *J. Agric. Food Chem.* **2022,** *70*, (14), 4457-4466.
52. Izquierdo-Sandoval, D.; Fabregat-Safont, D.; Lacalle-Bergeron, L.; Sancho, J. V.; Hernandez, F.; Portoles, T., Benefits of Ion Mobility Separation in GC-APCI-HRMS Screening: From the Construction of a CCS Library to the Application to Real-World Samples. *Anal. Chem.* **2022,** *94*, (25), 9040-9047.
53. Hernandez-Mesa, M.; D'Atri, V.; Barknowitz, G.; Fanuel, M.; Pezzatti, J.; Dreolin, N.; Ropartz, D.; Monteau, F.; Vigneau, E.; Rudaz, S.; Stead, S.; Rogniaux, H.; Guillarme, D.; Dervilly, G.; Le Bizec, B.,

Interlaboratory and Interplatform Study of Steroids Collision Cross Section by Traveling Wave Ion Mobility Spectrometry. *Anal. Chem.* **2020,** *92*, (7), 5013-5022.
54. Plachka, K.; Pezzatti, J.; Musenga, A.; Nicoli, R.; Kuuranne, T.; Rudaz, S.; Novakova, L.; Guillarme, D., Ion mobility-high resolution mass spectrometry in anti-doping analysis. Part I: Implementation of a screening method with the assessment of a library of substances prohibited in sports. *Anal. Chim. Acta* **2021,** *1152*, 338257.
55. Padron, J.; Carrasco, R.; Pellon, R., Molecular descriptor based on a molar refractivity partition using Randic-type graph-theoretical invariant. *J. Pharm. Pharmaceut. Sci.* **2002,** *5*, (3), 258-266.
